# USP21 deubiquitinates and stabilizes HSP90 and ENO1 to promote aerobic glycolysis and proliferation in cholangiocarcinoma

**DOI:** 10.7150/ijbs.90774

**Published:** 2024-02-04

**Authors:** Xiao Xu, Yananlan Chen, Shenye Shao, Jifei Wang, Jijun Shan, Yuming Wang, Yirui Wang, Jiang Chang, Tao Zhou, Ruixiang Chen, Shuochen Liu, Chang Li, Changxian Li, Xiangcheng Li

**Affiliations:** 1 Hepatobiliary Center, The First Affiliated Hospital of Nanjing Medical University, Nanjing, Jiangsu Province, China.; 2 Key Laboratory for Liver Transplantation, Chinese Academy of Medical Sciences, NHC Key Laboratory of Living Donor Liver Transplantation (Nanjing Medical University), Nanjing, Jiangsu Province, China.; 3 The Affiliated Wuxi People's Hospital of Nanjing Medical University, Wuxi People's Hospital, Wuxi Medical Center, Nanjing Medical University, Nanjing, Jiangsu Province, China.

## Abstract

Deubiquitylating enzymes (DUBs) play an essential role in targeted protein degradation and represent an emerging therapeutic paradigm in cancer. However, their therapeutic potential in cholangiocarcinoma (CCA) has not been explored. Herein, based on The Cancer Genome Atlas (TCGA) and The Gene Expression Omnibus (GEO) databases, we found that ubiquitin-specific protease 21 (USP21) was upregulated in CCA, high USP21 level was associated with poor prognosis. In vivo and in vitro, we identified USP21 as a master regulator of CCA growth and maintenance, which directly interacted with deubiquitinates and stabilized the heat shock protein 90 (HSP90) through K48-linked deubiquitination, and in turn, this stabilization increased HIF1A expression, thus upregulating key glycolytic enzyme genes ENO2, ENO3, ALDOC, ACSS2, and then promoted aerobic glycolysis, which provided energy for CCA cell proliferation. In addition, USP21 could directly stabilize alpha-Enolase 1 (ENO1) to promote aerobic glycolysis. Furthermore, increased USP21 level enhanced chemotherapy resistance to the gemcitabine-based regimen. Taken together, we identify a USP21-regulated aerobic glycolysis mechanism that involves the USP21/HSP90/HIF1A axis and USP21/ENO1 axis in CCA tumorigenesis, which could serve as a potential target for the treatment of CCA.

## Introduction

Cholangiocarcinoma (CCA) is an epithelial cell malignancy arising from varying locations within the biliary tree. The most contemporary classification based on anatomical location includes intrahepatic (ICC), perihilar (pCCA), and distal (dCCA) cholangiocarcinoma^1^. The global mortality for CCA increased worldwide during the past 20 years, and the incidence and mortality of CCA in Asian countries/regions are higher than those in Western countries/regions^2^, ^3^. This tumor frequently presents as sporadic cancer in patients without defined risk factors and is usually diagnosed at advanced stages with a consequent poor prognosis^4^. The mortality rate of CCA is almost equal to the morbidity due to the resistance of common treatments and the high recurrence rate after surgery^5^. Therefore, it is particularly important to study the molecular and cellular mechanisms of CCA progression and identify feasible and applicable therapeutic approaches.

The human genome encodes some 100 Deubiquitylating enzymes (DUBs), of which five sub-types are characterized as cysteine peptidases comprising USPs (ubiquitin-specific proteases), UCHs (ubiquitin carboxy-terminal hydrolases), MJDs (Machado-Josephin domain-containing proteases), OTUs (ovarian tumor proteases) and MINDYs (motif-interacting with ubiquitin-containing novel DUB family)^6^. In recent years, more and more evidence has shown that dysregulation of DUBs is related to the progression of human tumor diseases^7^. Ubiquitin-specific protease 21 (USP21) is a member of the USPs, which is the largest and most structurally diverse family of DUBs. By screening TCGA, GEO, and the center databases, it was found that USP21 was highly expressed in CCA and associated with poor prognosis. Previous studies have found that USP21 is closely related to the occurrence and development of tumors. In pancreatic cancer, USP21 activation promotes tumor growth via Wnt pathway activation^8^. In addition, USP21 has also been reported to promote tumorigenicity in mesenchymal glioblastoma stem cells by deubiquitinating FOXD1^9^. Although it has been reported that USP21 expression is upregulated in cholangiocarcinoma^10^, the mechanism of USP21 in the progression of CCA has not been studied.

In this study, we provide the first clarification of the molecular mechanism and signaling pathway of USP21 in CCA. We found that USP21 plays a key role in the regulation of tumor aerobic glycolysis. Upregulation of USP21 stabilizes HIF1A through deubiquitination of HSP90, ultimately leading to upregulation of key glycolytic enzymes. Moreover, USP21 could directly stabilize ENO1 to promote aerobic glycolysis. In conclusion, our study suggests that USP21 plays an important role in the progression of CCA; Therefore, the USP21/HSP90/HIF1A axis and USP21/ENO1 axis may serve as potential targets for molecular-based therapy for CCA.

## Materials and Methods

### Human tissue samples and microarray

The tissue microarray was constructed in Outdo Biotech Company (Shanghai, China) from 210 CCA patients. All of the patients underwent surgery at the First Affiliated Hospital of Nanjing Medical University between 2006 and 2017. The expression level of USP21 was calculated by the semi-quantitative scoring system. The intensity of staining was classified in four degrees including negative (0), weak (1), moderate (2), or strong (3), and the percentage of positive cells in the staining tissues was scored as follows: 0-5% (0), 6-35% (1), 36-70% (2), >70% (3). The overall score was calculated by multiplying the score of intensity and percentage. Two independent pathologists perform staining scores for each microarray tissue, with the average score as the final score. The score of tumor tissues ≥4 was considered as upregulated, otherwise considered as downregulated.

The patients were followed up regularly until their death or October 25, 2019. The use of clinical samples was approved by the Ethics Committee of The Affiliated Hospital of Nanjing Medical University. Informed consent was obtained in accordance with regional regulations.

### Immunohistochemistry (IHC) and immunofluorescence (IF) assay

Microarrays of cholangiocarcinoma tissues and sections of mice xenografts were prepared for immunohistochemical staining. The slides were immersed in 3% H2O2 for 5 min at room temperature to block endogenous peroxidase activity and incubated in sodium citrate buffer for 15 min at 95°C for antigen retrieval. After being blocked with 5% normal goat serum for 10 min, the slides were incubated with corresponding antibodies overnight at 4°C, followed by incubation with appropriate secondary antibody for 1 hour at room temperature. Nuclei were visualized by DAPI (Beyotime, China) staining. The images were taken through a fluorescence microscope (Olympus, Japan).

For immunofluorescence, cells attached to slides were fixed with 4% paraformaldehyde and permeabilized with Immunostaining Permeabilization Buffer with Saponin (Beyotime, China). After washing, the sliders were blocked with 5% BSA in PBS for 1 hour at room temperature and then incubated with appropriate primary antibody overnight at 4°C. The cells were washed three times and incubated with the corresponding secondary antibody for 1 hour at room temperature. Nuclei were stained with DAPI. The slides were photographed under a fluorescence microscope (Olympus, Japan).

### RNA sequencing

Total RNA was extracted from HuCCT1 cells in the negative control and Si-USP21 groups using TRIzol reagent (Invitrogen, California, USA). The purity and concentration of total RNA were measured by NanoDrop 2000 nucleic acid protein analyzer (Thermo Scientific, MA, USA). RNA-seq analysis was performed by Outdo Biotech Company (Shanghai, China).

### IP coupled with mass spectrometry

Total proteins were extracted from CCA cells and IP was performed with the USP21 antibodies (#sc-515911, Santa Cruz Biotechnology) and Protein A/G-agarose beads (Thermo Scientific, MA, USA). The mass spectrometry analyses were carried out by BGI Tech Solutions Co., Ltd (BGI Shenzhen, Guangdong, China).

### Statistical analysis

All statistical analyses were performed with SPSS v24.0 (IBM, SPSS, Chicago, IL, USA) and GraphPad Prism 7 (GraphPad Software, La Jolla, USA). Differences between the two groups were analyzed by Student's t-test. Correlations between USP21 expression and clinicopathological variables were analyzed by χ^2^ test. The Kaplan-Meier methods and the log-rank test were applied for estimating OS and DFS. The Cox proportional hazard regression model was used for multivariate analysis. Correlations between USP21 and downstream genes were tested by Pearson rank correlation analysis. When *p < 0.05, **p < 0.01, or ***p < 0.001, the difference is considered statistically significant.

Further materials and methods are provided in the supplementary materials and methods.

## Results

### USP21 was upregulated in CCA tissues and associated with poor prognosis

**Figure 1:**
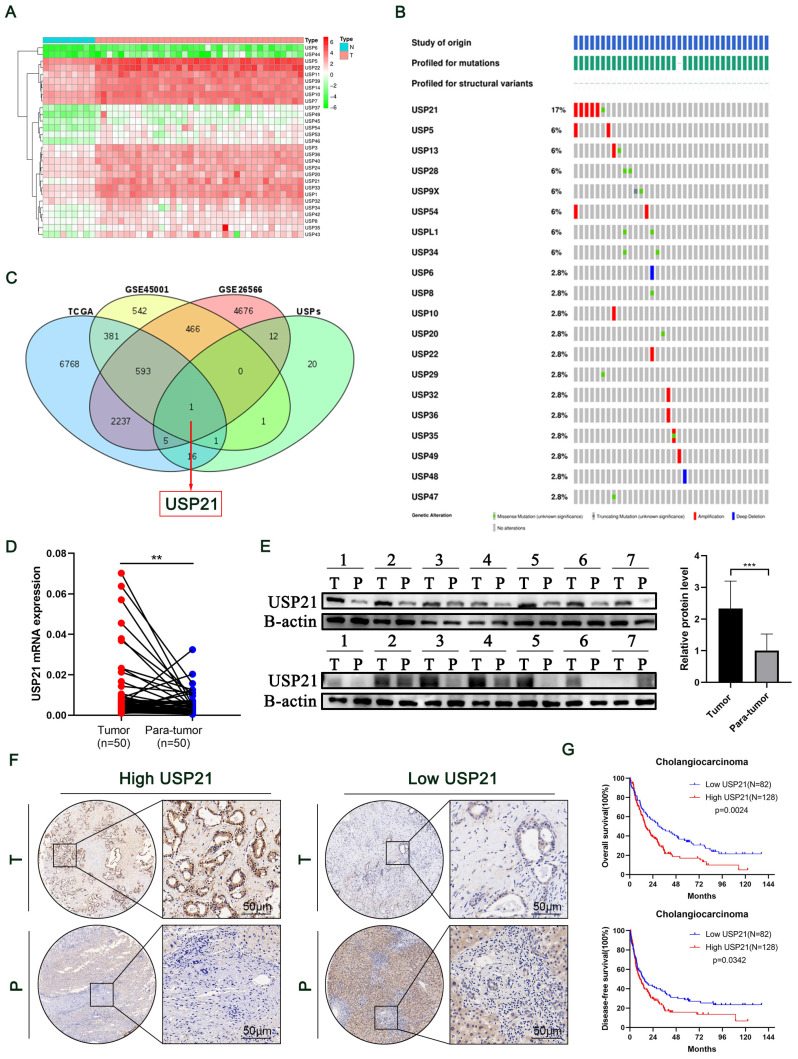
** USP21 was up-regulated in CCA tissues and associated with poor prognosis.** (A) Heat map of differentially expressed genes in the USP family in TCGA dataset. (B) Genetic alternations of USPs in TCGA CCA dataset. (C) Differentially expressed genes were searched by TCGA, GSE45001, and GSE26566 datasets. (D) RT-qPCR analysis of USP21 expression in CCA tissues and paired non-tumor tissues. (E) Western blotting analysis of USP21 expression in CCA tissues and paired non-tumor tissues. (F) Representative images of high/low expression of USP21 in tumors and their paired non-tumor tissues were shown, and the scores were calculated by the intensity and percentage of stained cells. (G) Upregulation of USP21 in CCA was correlated with poor OS and DFS after surgery. *P < 0.05, **P < 0.01, ***P < 0.001.

Humans have more than 50 USP family deubiquitinating enzymes, making this class of DUBs the largest^11^. To explore their function in CCA, we drew a heat map of differentially expressed genes based on the TCGA dataset (Figure 1A). Then, Genomic analysis of all the USPs in human CCA samples micro-dissected for cancer cell enrichment revealed that USP21 had the highest amplification rate in all cases (Figure 1B)^12^. To identify key genes that contributed to the development of CCA in the USP family, we combined TCGA and GEO databases (GSE26566, GSE45001) to probe upregulated genes and set uniform criteria in three datasets (Log2FC>1, P<0.01). Ultimately, USP21 was confirmed to be the only gene that was simultaneously highly expressed in all three datasets (Figure 1C; Supplementary Figure 1A-C). Then, we further verified that USP21 was highly expressed in CCA tissues in the GES107943 dataset (Supplementary Figure 1D). Previous reports suggested that USP21 regulates cell proliferation in hepatocellular carcinoma^13^, however, there were no molecular mechanistic insights as to whether and how USP21 may contribute to CCA oncogenesis, thus, we chose USP21 for further research. RT-qPCR was performed to explore the mRNA expression levels of USP21 in 50 randomly selected patients diagnosed with CCA from our center. Compared with that in paired non-tumor tissues, the USP21 mRNA expression level was significantly upregulated in tumor tissues (Figure 1D). Subsequent examination by western blotting in randomly selected 14 pairs of samples showed that the USP21 protein level was upregulated in tumor tissues more than that of paired non-tumor tissues (Figure 1E).

To further determine the clinical significance of USP21 in CCA progression, immunohistochemical staining (IHC) was performed in CCA tissue microarray (TAM). The representative images of high/low expression levels of USP21 were revealed in Figure 1F. Next, we analyzed the correlation between USP21 protein expression and clinicopathological features of CCA, as detailed in Table 1. USP21 protein expression was significantly correlated with tumor location (p=0.034) and perineural invasion (p=0.002). Univariate analysis showed that USP21 expression, patient's gender, tumor size, tumor differentiation, N stage, and surgical margin were significantly correlated with OS in CCA patients. In addition, multivariate analysis indicated that USP21 expression, tumor size, and N stage were independent risk factors for postoperative OS (Table 2). Kaplan-Meier survival curves showed that the group with high USP21 expression had a lower overall survival rate (P=0.0014) and a lower disease-free survival rate (P=0.0342) than the group with low USP21 expression (Figure 1G). Together, the results demonstrated that USP21 might be a competent indicator for CCA progression and patients' survival after surgery.

### USP21 promoted the proliferation of CCA in vitro and in vivo

To select appropriate USP21 knockdown and overexpression CCA cell lines, we detected the mRNA and protein expression levels of USP21 in CCA cell lines by RT-qPCR and Western bolt. Compared with normal bile ducts epithelial, USP21 mRNA and protein expression levels were much higher in all four CCA cell lines, among which, USP21 expression level was the highest in QBC939 and relatively low in HuCCT1(Supplementary Figure 2A-B). Therefore, we selected QBC939 cells to silence USP21 using siRNA, and HuCCT1 cells to increase USP21 level by lentivirus. The transfected efficiency of USP21 was validated both in mRNA and protein levels (Supplementary Figure 2C-D).

**Figure 2:**
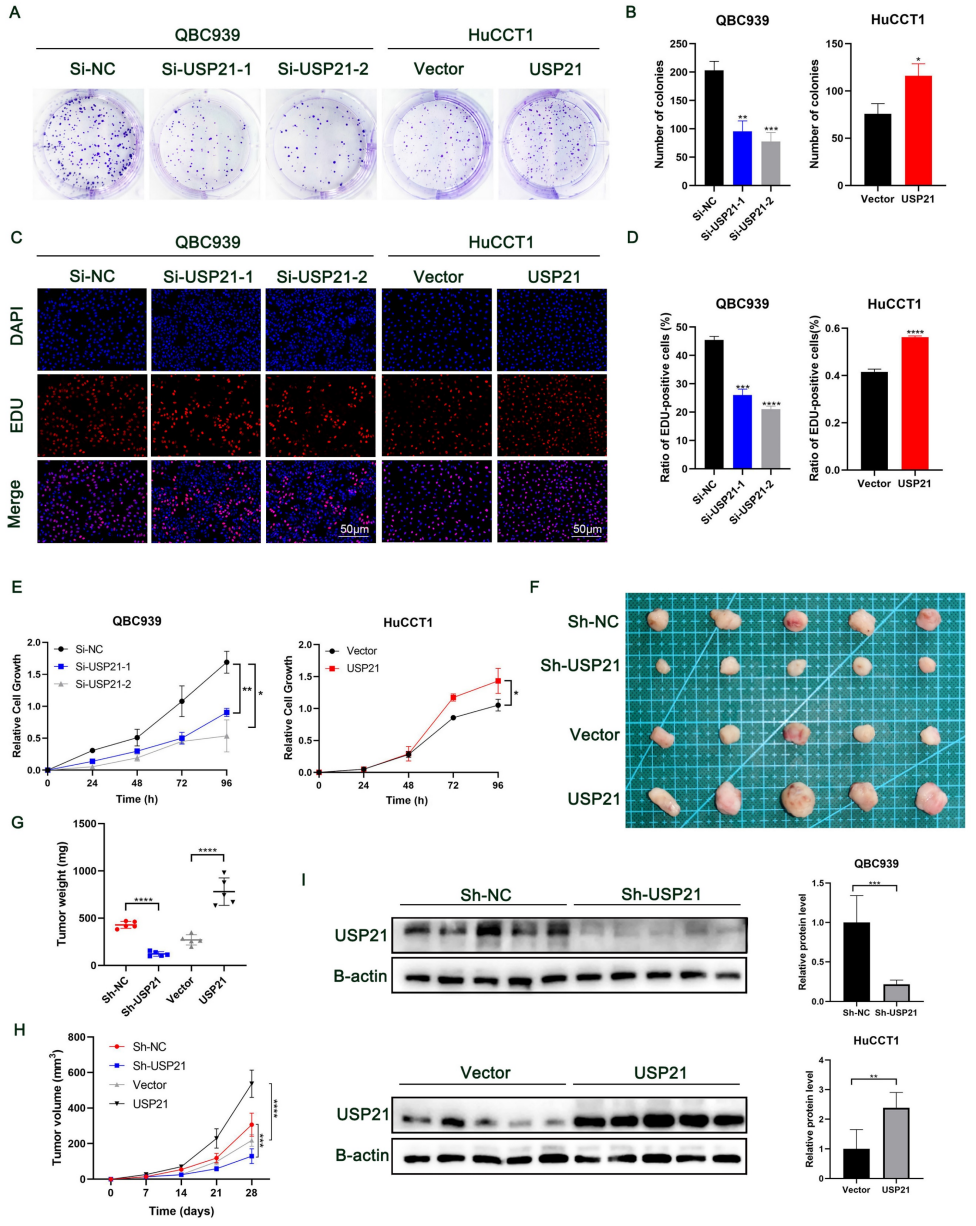
** USP21 promoted the proliferation of CCA in vitro and in vivo.** (A-B) Plate clone formation assays, (C-D) EDU staining assays, and (E) CCK8 assays indicated that USP21 promoted the proliferation of CCA cells. (F) Representative images showed the tumors removed from nude mice subcutaneously injected with USP21 knockdown or overexpression cells. (G-H) The quantification of tumor weight and tumor volume. (I) The efficiency of USP21 knockdown or overexpression in vivo was evaluated by western blotting. *P < 0.05, **P < 0.01, ***P < 0.001.

Plate clone formation assays showed that low expression of USP21 significantly inhibited the proliferation of CCA cells, while overexpression of USP21 enhanced the proliferation of CCA cells (Figure 2A-B). Next, more EDU-positive cells were observed in USP21 overexpression cells compared to the USP21 knockdown cells by EDU assay (Figure 2C-D). Additionally, the CCK-8 assay showed that USP21 knockdown suppressed the cell viability of CCA while the cell viability in the USP21 overexpression group was significantly increased (Figure 2E). Then, we used RBE cells to knockdown or overexpression USP21 to further verify the function of USP21 in CCA. CCK8 assays, plate clone formation assays, and EDU staining assays eventually demonstrated the same trend (Supplementary Figure 2E-G). These results suggested that USP21 can promote CCA cell proliferation in vitro. Subsequently, we conducted in vivo experiments to assess the effect of USP21 on tumorigenesis. QBC939 cells with stably transfected sh-USP21 and the corresponding control sh-NC and HuCCT1 cells with stable USP21 overexpression and the corresponding control Vector were inoculated subcutaneously into nude mice. Tumors were harvested 28 days after inoculation (Figure 2F). Notably, the weight and volume of tumors with USP21 knockdown cells were evidently less than that with control QBC939 cells. In line with that, xenografts derived from USP21 overexpression HuCCT1 cells were larger and heavier than those from control HuCCT1 cells (Figure 2G-H). Next, western blotting was used to evaluate the efficiency of USP21 knockdown or overexpression in vivo (Figure 2I). In conclusion, we demonstrated that USP21 promoted CCA proliferation in vivo.

### USP21 promoted the proliferation of CCA cells by enhancing aerobic glycolysis through the HIF-1 signaling pathway

**Figure 3:**
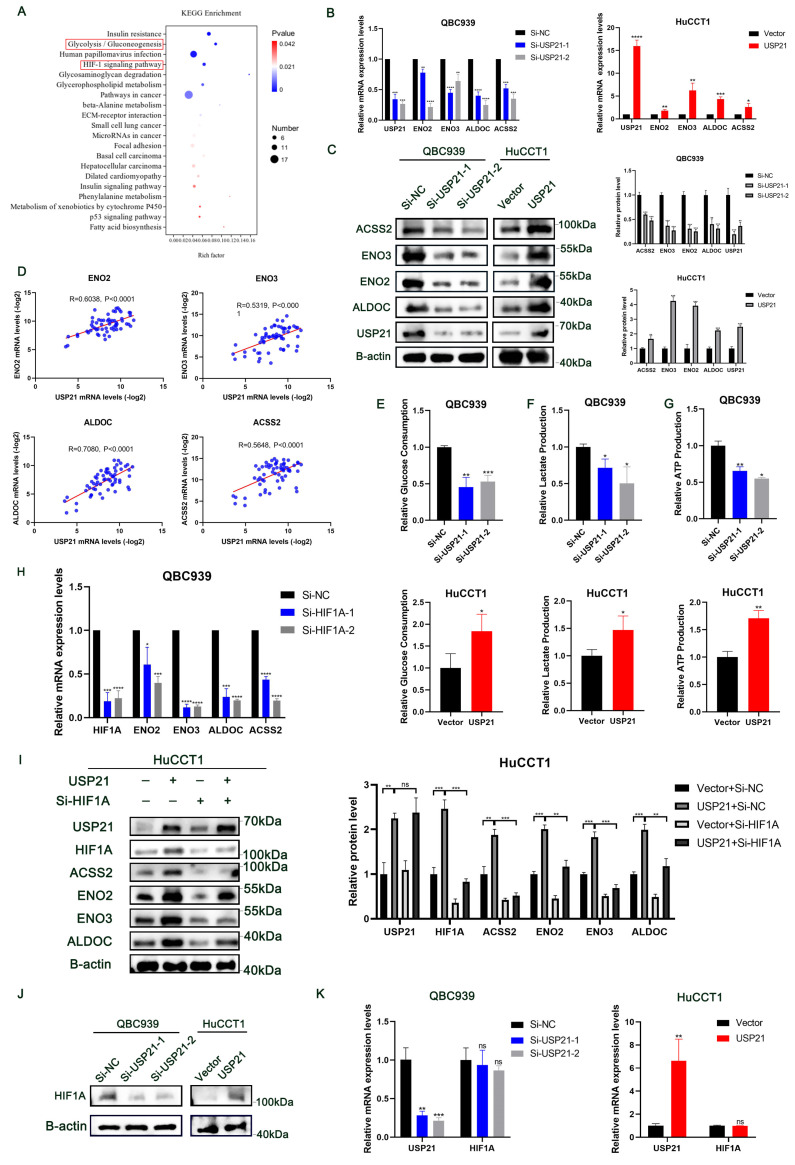
** USP21 promoted the progression of CCA by enhancing aerobic glycolysis through the HIF-1 signaling pathway.** (A) KEGG pathway analysis of downregulated genes in USP21 knockdown QBC939 cells. (B) RT-qPCR analysis of mRNA expression of glycolytic enzyme genes ENO2, ENO3, ALDOC, and ACSS2 in USP21 knockdown and overexpression CCA cells. (C) Protein expression levels of glycolytic enzyme genes were determined by western blotting in USP21 knockdown QBC939 cells and USP21 overexpression HuCCT1 cells. (D) Scatter plot analysis of the correlation between mRNA levels of USP21 and ENO2, ENO3, ALDOC, and ASCC2 in CCA tissues. (E-F) USP21 knockdown and USP21 overexpression CCA cells were treated with DMEM without serum for 24 h. The media were collected for analysis of glucose consumption and lactate production. n = 3 for each group. (G) The indicated cells were cultured for 24 h under aerobic conditions. ATP levels in cellular supernatants were determined by chemiluminescence. n = 3 for each group. (H) RT-qPCR analysis of mRNA expression of ENO2, ENO3, ALDOC, and ACSS2 in HIF1A knockdown CCA cells. (I) Knockdown of HIF1A in HuCCT1 cells stably overexpressing USP21 significantly reversed the up-regulation of ENO2, ENO3, ALDOC, and ACSS2 protein expression levels. (J-K) The relative mRNA and protein expression levels of HIF1A were examined by western blotting and RT-qPCR. *P < 0.05, **P < 0.01, ***P < 0.001.

To further explore the molecular mechanism of USP21 involved in CCA progression, RNA sequencing analysis of QBC939 treated with Si-NC or Si-USP21 was performed to determine gene expression changes (Supplementary Figure 3A). The KEGG enrichment analysis showed that USP21 knockdown affected a variety of signaling pathways related to tumor and metabolism in CCA cells, among which Glycolysis/Gluconeogenesis and HIF-1 signaling pathway scored significantly higher than other pathways (Figure 3A). GO enrichment also revealed multiple biosynthetic processes (Supplementary Figure 3B). Next, we selected the most differentially expressed genes ENO2, ENO3, ALDOC, and ACSS2 from the Glycolysis/Gluconeogenesis pathway according to the sequencing analysis results, and verified their mRNA expression in USP21 knockdown and overexpression CCA cells by RT-qPCR. Consistent with the sequencing analysis results, the mRNA levels of ENO2, ENO3, ALDOC, and ACSS2 were significantly decreased in USP21 knockdown QBC939 cells, while increased in USP21 overexpression HuCCT1 cells (Figure 3B). Western blotting results also confirmed that USP21 could regulate glycolytic enzyme synthesis (Figure 3C). To further validate our results, we examined the mRNA levels of USP21, ENO2, ENO3, ALDOC, and ACSS2 in 60 randomly selected CCA tissue samples. The Pearson Correlation analysis showed a positive correlation between USP21 and ENO2 (r=0.6038, p<0.0001), ENO3 (r=0.5319, p<0.0001), ALDOC (r=0.7080, p<0.0001), and ACSS2 (r=0.5648, p<0.0001) (Figure 3D).

Moreover, we explored the effects of USP21 on aerobic glycolysis in CCA cells. USP21 knockdown inhibited the glycolytic capacity of QBC939 cells, further reducing glucose consumption, lactate production, and cellular ATP level. In contrast, glucose consumption, lactate production, and cellular ATP production were increased in HuCCT1 cells stably overexpressing USP21 (Figure 3E-G). Aberrant glycolysis is frequently associated with tumorigenesis and cancer progression. Thus, we hypothesized that USP21-mediated enhancement of glycolysis plays a role in the proliferation of CCA cells. We knocked down ENO2, ENO3, ALDOC, and ACSS2 in HuCCT1 cells stably overexpressing USP21. As is shown in Supplementary Figure 3C-E, the knockdown of glucose metabolic genes reversed the increase in glucose consumption, lactate production, and cellular ATP production caused by USP21 overexpression. Colony formation assays, EDU staining assays, and CCK8 assays further indicated that glucose metabolic genes are indispensable for the proliferative regulatory function of USP21 in CCA cells (Supplementary Figure 3F-H).

HIF1A is a critical oncogenic factor in the HIF-1 signaling pathway. According to previous studies, HIF1A, a transcriptional activator, is closely related to aerobic glycolysis in tumor cells^14^, ^15^, and was positively correlated with the expression of ENO2, ENO3, ALDOC, and ACSS2^16^-^18^. The results of RT-qPCR further verified that HIF1A could regulate the mRNA expression of glucose metabolic genes in CCA cells (Figure 3H). In addition, the knockdown of HIF1A in HuCCT1 cells stably overexpressing USP21 significantly reversed the up-regulation of ENO2, ENO3, ALDOC, and ACSS2 protein expression levels (Figure 3I). Likewise, functional experiments confirmed that USP21 promoted tumor growth in a HIF1A-dependent manner (Supplementary Figure 4A-C). To further elucidate the underlying mechanism of USP21-meditated upregulation of the glucose metabolic genes, we then measured the mRNA and protein level of HIF1A in USP21 knockdown and overexpression cells. Interestingly, the protein expression of HIF1A was positively correlated with USP21 (Figure 3J, Supplementary Figure 4D), while the mRNA expression level did not change significantly (Figure 3K). Based on the above results, we hypothesized that USP21 altered the transcription of glucose metabolic genes by regulating the total protein expression of HIF1A. Collectively, these results suggested that USP21 may promote the proliferation of CCA cells by enhancing aerobic glycolysis through the HIF-1 signaling pathway.

### USP21 interacted with HSP90 and ENO1

**Figure 4:**
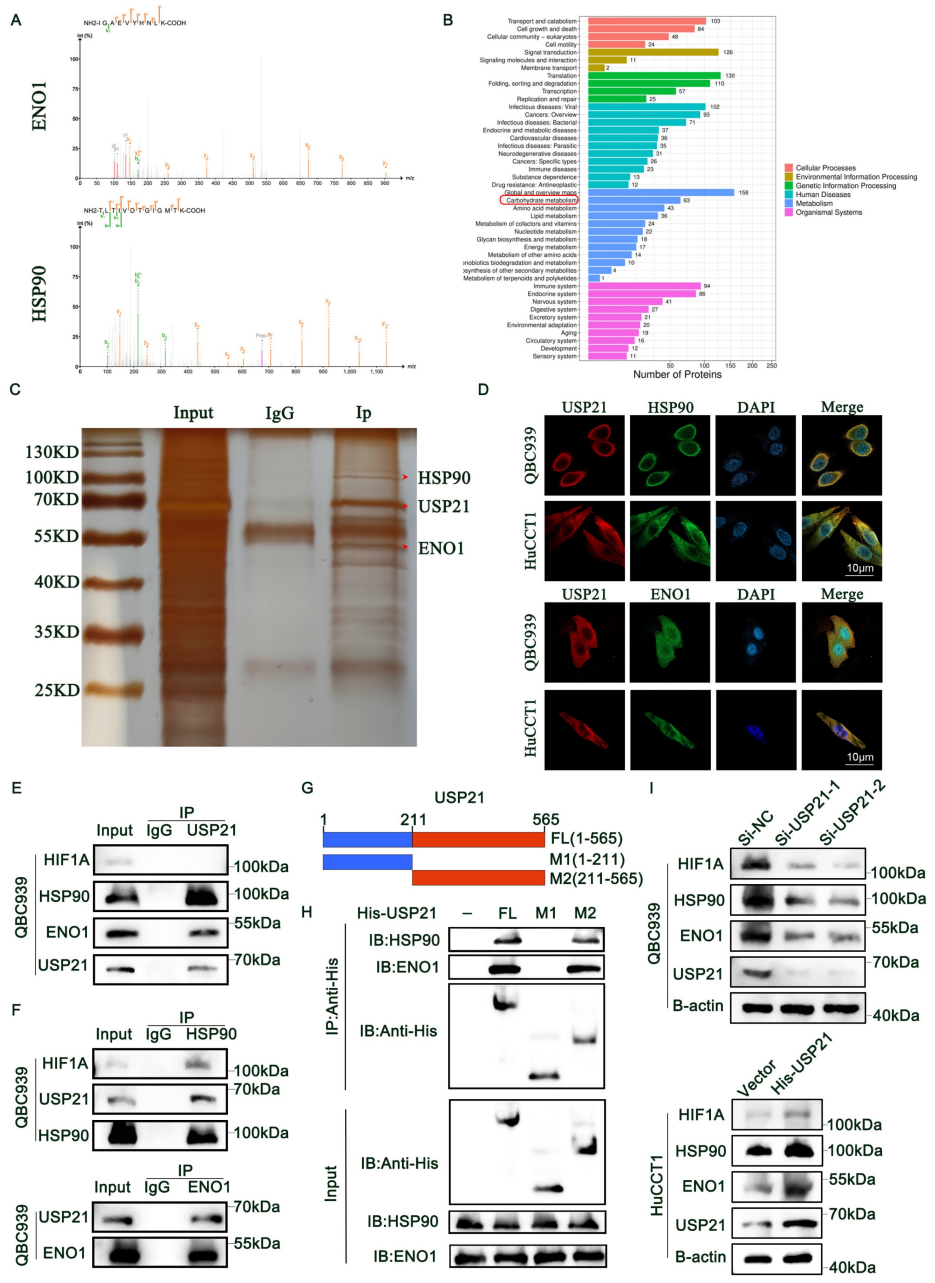
** USP21 interacted with HSP90 and ENO1.** (A) IP/MS analysis was used to determine which proteins bind USP21, thus identifying HSP90 and ENO1 as interacting proteins. (B) KEGG pathway analysis of IP/MS analysis results. (C) The sensitive silver staining method was used to find the interacting proteins. (D) Immunofluorescence (IF) assays verified the binding of USP21 with HSP90 and ENO1 in CCA cells. (E) Co-IP was performed to confirm the binding of HSP90 and ENO1 to USP21 in QBC939 cells. (F) Immunoprecipitation assays were performed in QBC939 cells to examine the endogenic interaction among HIF1A, HSP90, ENO1, and USP21. (G) Schematic structures of USP21, together with their truncated mutants. (H) Co-IP showed that the C terminal of USP21 is essential for the interaction with HSP90 and ENO1. (I) Western blotting analysis was used to detect the expression of HIF1A, HSP90, and ENO1 in USP21 knockdown and overexpression CCA cells.

To get a deeper insight into the mechanism by which USP21 regulates HIF1A protein expression in CCA cells. IP coupled with MS (IP/MS) was used to detect the proteins binding to USP21. Based on the analysis results, we identified heat-shock protein 90 (HSP90), which acts as a molecular chaperone and is reported to directly regulate the degradation of HIF1A^19^, ^20^. Herein, 17-AAG, an HSP90 inhibitor, was adopted to inhibit HSP90 expression in CCA cells. Notably, we found that decreased HSP90 expression level can effectively reduce the total protein expression of HIF1A (Supplementary Figure 5A). In addition, Enolase 1 (ENO1), a key enzyme in the Glycolysis/Gluconeogenesis pathway^21^, ^22^, was also found to bind to USP21 (Figure 4A). KEGG enrichment analysis further showed that the carbohydrate metabolism pathway was the significantly enriched pathway (Figure 4B). Then, we used a sensitive silver staining method to further confirm the bound proteins (Figure 4C). Immunofluorescence assays verified that USP21 interacted with HSP90 and ENO1 in CCA cells (Figure 4D). Next, co-IP analysis was performed to further confirm the IP/MS analysis results. As is shown in Figure 4E, USP21 precipitated HSP90 and ENO1 in QBC939 cells, while HIF1A was not precipitated. Consistent results were also detected in HuCCT1 cells (Supplementary Figure 5B). Reverse Co-IP confirmed that USP21 was significantly precipitated by HSP90 and ENO1 in CCA cells. We also found that HIF1A could also be precipitated by HSP90 (Figure 4F, Supplementary Figure 5C-D). USP21 is composed of an NH2-terminal domain, and a C-terminal USP domain (Figure 4G). To map the minimal essential domain required for USP21 to interact with HSP90 and ENO1, His-USP21 or its truncated mutants were transfected into HEK293T cells. Co-IP analysis showed that the C-terminal of USP21 is essential for the interaction with HSP90 and ENO1 (Figure 4H). Moreover, we observed that the altered HIF1A, HSP90, and ENO1 expression levels were coincident with the dynamic USP21 expression level (Figure 4I, Supplementary Figure 5E). Based on these results, we suggested that USP21 interacts directly with HSP90 and ENO1 and affects HIF1A expression levels through HSP90 in CCA cells.

### USP21 inhibited HSP90 and ENO1 ubiquitination and degradation in human CCA cells

**Figure 5:**
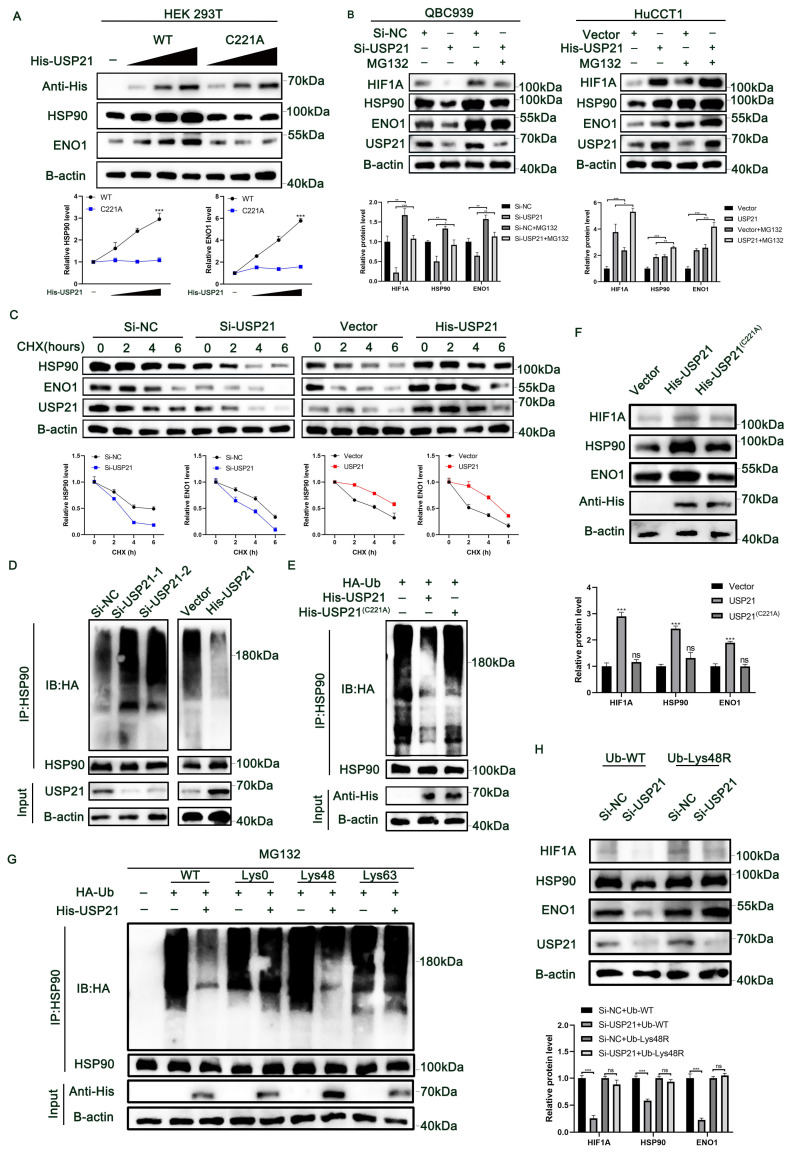
** USP21 inhibited HSP90 and ENO1 ubiquitination and degradation in human CCA cells.** (A) Increasing His-tagged USP21 (WT or C221A mutant) was transfected into HEK 293T cells, and the cell lysates were analyzed by western blotting with anti-HSP90 and anti-ENO1. (B) Analysis of HIF1A, HSP90, ENO1, and USP21 in USP21 knockdown and overexpression CCA cells with or without treatment with the proteasome inhibitor MG132. (C) HSP90 and ENO1 protein levels in USP21 knockdown and overexpression CCA cells were measured by western blotting in the absence and presence of cycloheximide (CHX, 10 μg/mL) for a specified time. (D) Lysates from CCA cells treated with MG132 before collecting were subjected to immunoprecipitation and detected with the indicated antibodies. (E) The lysates of HEK 293T cells transfected with HA-Ub as well as His-labeled USP21 (WT) or His-labeled USP21 (C221A), were immunoprecipitated and subjected to anti-HA and anti-HSP90 immunoblotting. (F) Analysis of HIF1A, HSP90, and ENO1 in CCA cells transfected with His-labeled USP21 (WT) or His-labeled USP21 (C221A). (G) CCA cells were co-transfected with His-USP21 and HA-Ub Lys0, Lys48 alone, or Lys63 alone plasmids, and then HSP90 ubiquitination was analyzed. (H) USP21 knockdown CCA cells were transfected with Ub WT or Ub Lys48R. Cell lysates were analyzed by immunoblotting with the indicated antibodies. *P < 0.05, **P < 0.01, ***P < 0.001.

RT-qPCR combined with RNA sequencing analysis results indicated that the regulation of HSP90 and ENO1 by USP21 does not occur at the mRNA level (Supplementary Figure 6A-B), but that the stability of HSP90 and ENO1 protein may be regulated by USP21. We then further investigated whether USP21 might stabilize HSP90 and ENO1. Overexpression of WT USP21 increased the protein levels of HSP90 and ENO1, whereas overexpression of the catalytically inactive C221A mutant USP21 did not cause such an increase (Figure 5A). The Addition of the proteasome inhibitor MG132 reversed the decrease in HSP90 and ENO1 protein levels after the USP21 knockdown (Figure 5B). We next studied the effect of USP21 knockdown or overexpression on the stability of endogenous HSP90 and ENO1 protein in the presence of the protein synthesis inhibitor cycloheximide. Compared with that in control CCA cells, the half-life of HSP90 and ENO1 proteins was shorter in USP21 knockdown CCA cells, whereas the opposite was observed in USP21 overexpression cells (Figure 5C). In addition, the half-life of HSP90 and ENO1 proteins did not change significantly in CCA cells transfected with USP21 (C221A) (Supplementary Figure 6C). Subsequently, we sought to determine whether USP21 might regulate the ubiquitination and degradation of HSP90 and ENO1. We transfected HA-Ub into CCA cells followed by the administration of MG132 to inhibit protein degradation. After co-immunoprecipitation with anti-Hsp90 and anti-ENO1 antibodies, it was observed that the ubiquitination level of HSP90 and ENO1 was increased in USP21 knockdown cells, while USP21 overexpression decreased the ubiquitination level (Figure 5D, Supplementary Figure 6D). To further confirm the effect of USP21 on HSP90 and ENO1 ubiquitination, we co-transfected HEK 293T cells with His-USP21 (WT or C221A mutant) and HA-Ub. Overexpression of WT USP21 abolished the ubiquitination of HSP90 and ENO1, while the C221A mutants had no such effect (Figure 5E, Supplementary Figure 6E). Besides, the protein expression levels of HIF1A, HSP90, and ENO1 ulteriorly validated the previous findings (Figure 5F). Then, we put focus on the effect of USP21 on the polyubiquitin modification of HSP90 protein and found that USP21 cleaves the polyubiquitin chain associated with Lys48 but has no significant effect on the polyubiquitin chain associated with Lys63 (Figure 5G). To confirm that Lys48-linked polyubiquitination is necessary for USP21-regulated HSP90 degradation, we expressed a Lys48-resistant (Lys48R) form of ubiquitin in USP21 knockdown cells and found that the expression of Lys48R ubiquitin eliminated the decrease in HSP90 and ENO1 induced by USP21 knockdown (Figure 5H). Based on the above findings, we suggest that USP21 inhibits ubiquitination and degradation of HSP90 and ENO1, deubiquitylates K48-linked polyubiquitylation of HSP90 and ENO1.

### USP21 promoted aerobic glycolysis and tumor growth in human CCA cells by increasing HSP90 and ENO1 levels

**Figure 6:**
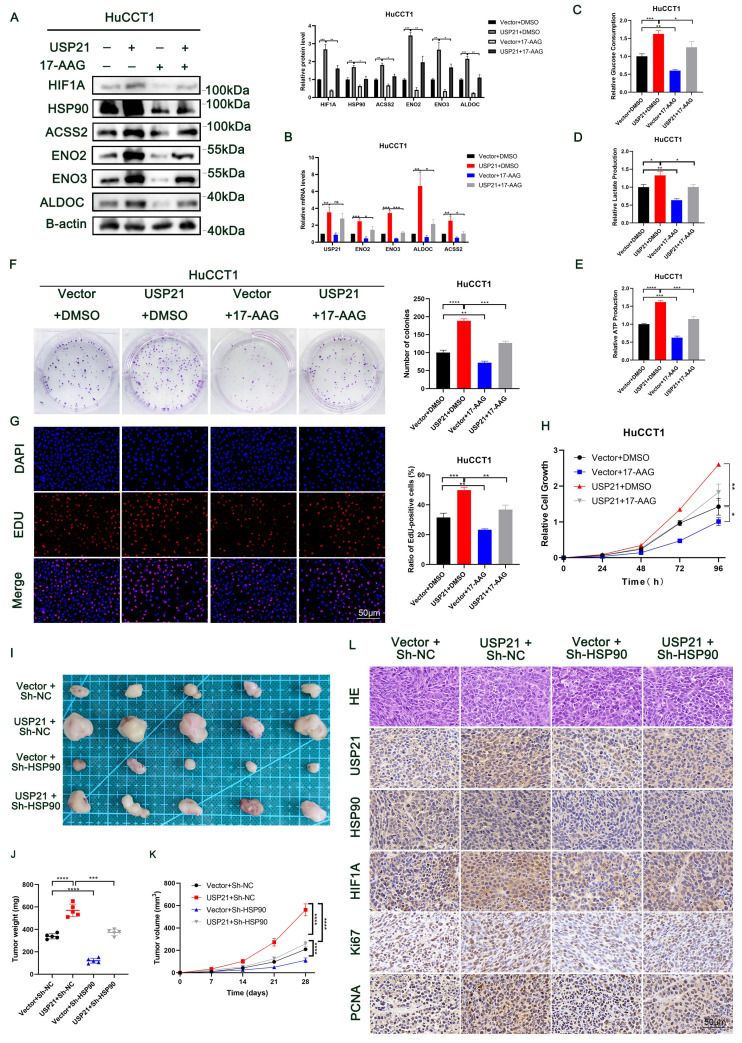
** USP21 promoted aerobic glycolysis and tumor growth in human CCA cells by increasing HSP90 and ENO1 levels.** HuCCT1 cells with USP21 overexpression were treated with 17-AAG at a dose of 50 nM, then the suppressive effect of glycolytic enzyme genes was verified by western blotting (A) and RT-qPCR (B). HSP90 inhibitor reversed USP21-mediated increases in glucose consumption (C), lactate production (D), and cellular ATP levels (E) in HuCCT1 cells. Functional assays containing Plate clone formation assays (F), EDU staining assays (G), and CCK8 assays (H) were performed to detect the rescued effect of HSP90 on USP21. (I) Xenograft tumors in nude mice revealed the rescued effect of HSP90 on USP21 in vivo. (J-K) The quantification of tumor weight and tumor volume. (L) The expression levels of USP21, HSP90, HIF1A, Ki67, and PCNA in nude mice-derived xenograft tumors were determined by IHC. *P < 0.05, **P < 0.01, ***P < 0.001.

To further verify the role of HSP90 in USP21-mediated CCA progress, we treated USP21 overexpression HuCCT1 cells with 17-AAG at a dose of 50 nM or DMSO in both groups. As shown in Figure 6A-B, in CCA cells, the HSP90 inhibitor significantly reversed the up-regulation of HIF1A protein expression caused by USP21 overexpression, and thus reversed the protein and mRNA expression levels of glycolytic enzyme genes ENO2, ENO3, ALDOC, and ACSS2. Next, we observed that the HSP90 inhibitor reversed USP21-mediated increases in glucose consumption, lactate production, and cellular ATP levels in HuCCT1 cells (Figure 6C-E). Functional experiments including plate clone formation assay, EDU staining assay, and CCK8 assay further verified the rescue effect of HSP90 inhibition on USP21 (Figure 6F-H). Subsequently, experiments in vivo were performed. The analysis based on the weight and volume of xenograft tumors showed that HSP90 knockdown partly diminished USP21-induced tumor growth (Figure 6I-K). Immunohistochemical staining of xenograft showed increased expression of proliferation markers Ki67 and PCNA in the USP21 overexpression group. We also found that the expression levels of HIF1A and glycolysis-related markers were higher in nude mouse tumors with USP21 overexpression, and HSP90 inhibition reversed this effect (Figure 6L, Supplementary Figure 7A).

In addition, we investigated the role of ENO1 in USP21-mediated CCA tumor progression. We knocked down ENO1 in HuCCT1 cells stably overexpressing USP21 (Supplementary Figure 7B). Similar to HSP90, ENO1 knockdown inhibited the glycolytic capacity of HuCCT1 cells (Supplementary Figure 7C-E), thus weakening the proliferation ability of HuCCT1 cells (Supplementary Figure 7F-H). All these results suggested that the mechanism by which USP21 regulates aerobic glycolysis and proliferation in CCA cells depends on the HSP90/HIF1A axis and the increased ENO1 expression.

### USP21 induces gemcitabine resistance in CCA cells

**Figure 7:**
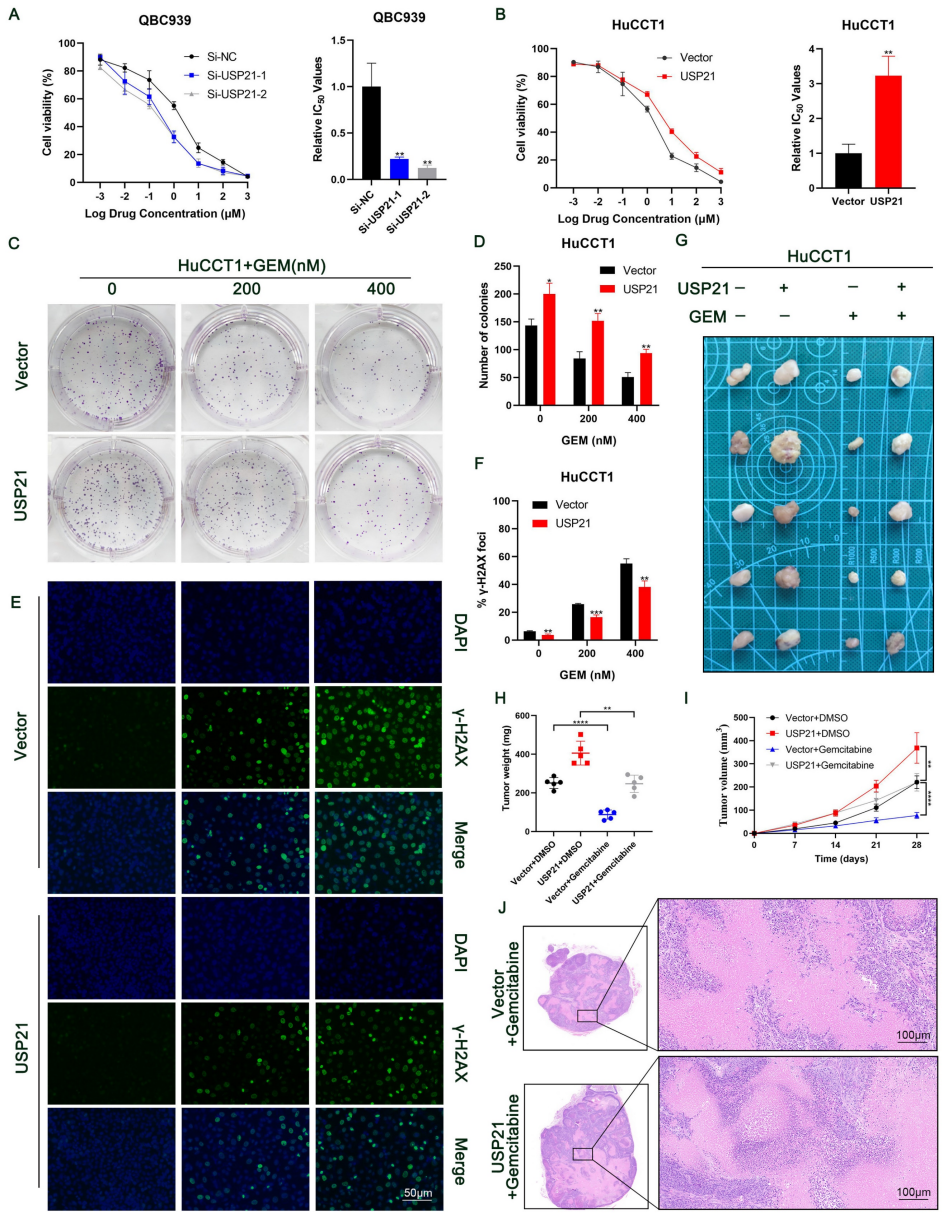
** USP21 induces gemcitabine resistance in CCA cells.** (A-B) GEM IC50 of QBC939 and HuCCT1 was determined by constructing a dose-response curve. (C-D) Colony formation assays of HuCCT1 cells treated with different doses of GEM. (E-F) Use of the γ-H2AX assays to monitor DNA damage in HuCCT1 cells treated with different doses of GEM. (G) HuCCT1 cells were subcutaneously injected into nude mice followed by GEM treatment (20 mg/kg) or not. (H-I) The quantification of tumor weight and tumor volume. (J) Representative images of H&E staining in xenograft tumors derived from gemcitabine-treated nude mice. *P < 0.05, **P < 0.01, ***P < 0.001.

Currently, gemcitabine-based chemotherapy is still the first-line treatment for advanced biliary tract tumors and has been shown to improve survival^23^, ^24^. Next, we explored whether USP21 affects the resistance of CCA cells to gemcitabine. CCK-8 assays showed that USP21 knockdown reduced the half-maximal inhibitory concentration (IC50) of gemcitabine (GEM) in CCA cells (Figure 7A). Conversely, upregulating USP21 induced GEM resistance in tumor cells (Figure 7B). Based on this, we then treated CCA cells with different doses of GEM (0/200/400nM). Colony formation assays showed that USP21 overexpression HuCCT1 cells had a higher survival rate after GEM treatment than the control cells (Figure 7C-D). γ-H2AX assay which indicates the degree of DNA damage in a single cell showed that USP21 overexpression reduced the number of γ-H2AX foci in HuCCT1 cells (Figure 7E-F). Subsequently, we further confirmed the role of USP21 in GEM resistance in CCA xenograft tumor models. HuCCT1 cells were injected subcutaneously into nude mice. When the tumor diameter reached 4 mm, 20 mg/kg GEM was injected intraperitoneally five times at 4-day intervals. Notably, the weight and volume of subcutaneous xenograft tumors in gemcitabine-treated mice significantly decreased compared with those without gemcitabine treatment. The efficacy of gemcitabine in the USP21 overexpression group was worse than that in the control group (Figure 7G-I). H&E staining in xenograft tumors derived from gemcitabine-treated nude mice showed that gemcitabine administration enhanced cell necrosis (Figure 7J). In conclusion, these results demonstrated that USP21 plays a pivotal role in gemcitabine resistance in CCA cells.

### High HSP90, HIF1A, and USP21 expression were associated with poor prognosis

**Figure 8:**
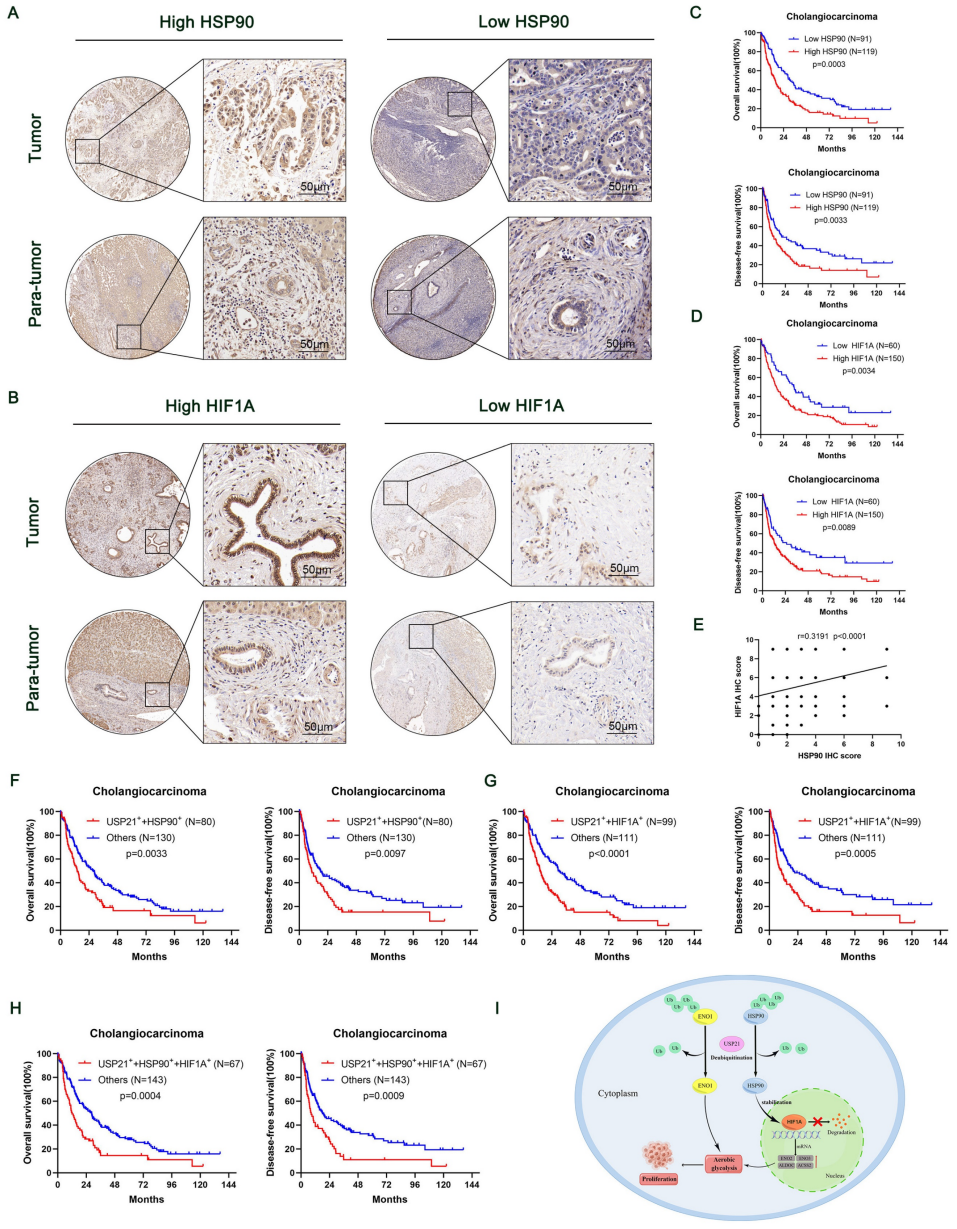
** High HSP90, HIF1A, and USP21 expression were associated with poor prognosis.** (A-B) Representative images of high/low expression of HSP90 and HIF1A in tumors and their corresponding para-tumor tissues were shown, and the scores were calculated by the intensity and percentage of stained cells. (C-D) Kaplan-Meier curves of overall survival (OS) and disease-free survival (RFS) in patients with high/low expression of HSP90 and HIF1A. (E) The correlation between HSP90 and HIF1A was analyzed based on the IHC score. (F-H) Kaplan-Meier survival curves showed OS and DFS in (USP21^+^ + HSP90^+^) group, (USP21^+^ + HIF1A^+^) group and (USP21^+^ + HSP90^+^ + HIF1A^+^) group based on IHC score. (I) Schematic representation of USP21-mediated aerobic glycolysis and proliferation in CCA cells.

To further reveal the contribution of HSP90 and HIF1A to the prognosis of USP21-positive CCA patients, we performed IHC on TMA containing 210 CCA patients (Figure 8A-B). The relationship between the clinicopathological characteristics of CCA patients and the protein expression levels of HSP90 and HIF1A was shown in Supplementary Table 1-4. Kaplan-Meier survival curves showed that high expression levels of HSP90 and HIF1A were associated with poor prognosis (Figure 8C-D). In addition, correlation analysis based on TMA IHC scores showed that HSP90 was significantly correlated with HIF1A in CCA tissues (Figure 8E). Further correlation analysis showed that the protein expression of HSP90 and HIF1A was positively correlated with that of USP21 (Supplementary Figure 8A, Supplementary Table 5). When USP21 was positively highly expressed with either HSP90 or HIF1A in patients with CCA, they had a much worse OS and DFS (Figure 8F-G). Moreover, we analyzed the triple correlation of USP21/HSP90/HIF1A and found that simultaneously high expression levels of USP21/HSP90/HIF1A suggested worse OS and DFS in CCA cohorts (Figure 8H). All these data suggested that USP21, HSP90, and HIF1A might be good prognostic indicators and therapeutic targets for CCA patients.

## Discussion

Accumulating evidence suggests that DUBs play a key role in tumor progression. Recently, Liao et al. [25]reported that USP1 regulates the growth and metastasis of human hepatoma cells by stabilizing the RPS16 protein. In addition, Li et al. [13]reported that USP21 promoted HCC tumor growth by stabilizing the MEK2 protein. Cholangiocarcinoma is the second most common primary hepatobiliary tumor after hepatocellular carcinoma. However, the molecular mechanism of DUBs on cholangiocarcinoma remains unclear.

In the current study, we report for the first time the role and mechanism of USP21 in CCA progression. In addition, we demonstrated a functional role for USP21 in regulating CCA cell glycolysis using the loss of function and gain of function approaches, which have not been previously reported. USP21 directly or indirectly promotes aerobic glycolysis of CCA cells by stabilizing HSP90 to regulate HIF1A protein degradation and stabilizing ENO1. Therefore, we believe that USP21 may be a promising therapeutic target for the treatment of CCA patients.

Increased glucose metabolism and reprogramming toward aerobic glycolysis are hallmarks of cancer cells that meet their metabolic demands for cell proliferation. Deregulation of metabolic pathways has been described during the onset and progression of CCA. Increased aerobic glycolysis allows CCA cells to generate biosynthetic intermediates^26^. From this perspective, a better understanding of aerobic glycolysis in CCA may help develop new strategies for treating this malignancy. Given this, we performed RNA sequencing and found that USP21 regulates specific genomes related to glucose metabolism. Then, our results further verified that USP21 could promote aerobic glycolysis in CCA cells through the HIF-1 pathway.

The activation of HIF1A is widely recognized as an essential marker for aerobic glycolysis^27^. Previous studies have demonstrated that HIF1A positively correlates with the expression of glycolytic enzymes, which promotes tumor proliferation and metastasis^14^, ^15^. To further investigate how USP21 regulates the HIF-1 pathway, we verified that USP21 could affect the expression of HIF1A at the protein level but not at the mRNA level. Furthermore, IP/MS was performed to determine that the HSP90 protein interacts with USP21 in CCA cells. HSP90 was the most reported function to act as a molecular chaperone. Studies have shown that in HEK293 cell and prostate cancer disease models, HSP90 and receptor for activated C kinase (RACK1) can competitively bind HIF1A, and then inhibit proteasomal pathway degradation^19^. Here, we verified that HSP90 interacted with HIF1A and stabilized HIF1A in CCA cells. We then hypothesized that activation of aerobic glycolysis by USP21 might occur through an HSP90-dependent mechanism. We also sought to determine whether USP21 could precipitate with antibodies to another protein, ENO1, which has been reported to be associated with aerobic glycolysis and has been validated by IP/MS. Our Co-IP results indicated that USP21 interacts with HSP90 and ENO1 in CCA cells. Meanwhile, the down-regulation of USP21 significantly decreased the protein level of HSP90 and ENO1, but not at its mRNA level, thus indicating that posttranslational modifications may play important roles in regulating the stability of HSP90 and ENO1. HSP90 and ENO1 have been widely reported to be regulated by the ubiquitin-proteasome system in various disease models^28^-^31^. We found that USP21 can stably interact with HSP90 and ENO1 and eliminate their ubiquitination, thereby reducing the degradation of HSP90 and ENO1 in CCA cells. Next, we performed in vitro and in vivo experiments to confirm that inhibition of HSP90 reversed the enhancement of aerobic glycolysis mediated by upregulation of HIF1A protein expression caused by USP21 overexpression. These data suggest that USP21 may regulate CCA cell progression by affecting the stability of HSP90 and ENO1. However, the specific binding sites of USP21 to HSP90 and ENO1 remain to be further explored. This is also one of the limitations of our study. In addition, the mechanism by which USP21 regulates CCA cell invasion is also a question to be addressed in the future.

Taken together, USP21-driven aerobic glycolysis may be related to the HSP90/HIF1A complex in CCA cells or the direct stabilization of ENO1. In this study, we demonstrate that USP21 stabilizes HSP90 and ENO1 through deubiquitination. Furthermore, patients with cholangiocarcinoma had poor prognoses and overall survival regardless of whether USP21 was highly expressed simultaneously with HSP90 or HIF1A. In summary, our findings highlight that USP21-mediated post-translational regulation of HSP90 and ENO1 is important in aerobic glycolysis and CCA tumor progression and provide an option for cancer therapy targeting glucose metabolism.

## Supplementary Material

Supplementary figures and tables.

## Figures and Tables

**Table 1:** Association of USP21 expression with clinicopathological features of CCA.

Clinicopathological features	USP21 expression		P value	χ2
	Low	High		
**All cases**	81 (38.6%)	129 (61.4%)		
**Gender**			0.892	0.018
Male	52 (64.2%)	84 (65.1%)		
Female	29 (35.8%)	45 (34.9)		
**Age**			0.579	0.308
≤60	44(54.3%)	65(50.4%)		
>60	37(45.7%)	64(49.6%)		
**Diameter(cm)**			0.597	0.280
≤2.5	19(23.5%)	29(22.5%)		
>2.5	43(53.0%)	79(61.2%)		
**Location**			**0.034**	**4.506**
Intrahepatic	49(60.5%)	57(44.1%)		
Perihilar	30(37.0)	65(50.4%)		
**Histological grade**			0.816	0.054
I/I-II/II	39(48.1%)	57(44.2%)		
II-III/III	39(48.1%)	61(47.3%)		
**Perineural invasion**			**0.002**	**9.859**
Absent	46(56.8%)	42(32.6%)		
Present	28(34.6%)	67(51.9%)		
**Tumor thrombus**			0.425	0.636
Absent	67(82.7%)	102(79.1%)		
Present	11(13.6%)	23(17.8%)		
**T stage**			0.113	2.514
Tis-T1	34(42.0%)	40(31.0%)		
T2-T4	44(54.3%)	83(68.6%)		
**N stage**			0.249	1.326
N0	52(64.2%)	91(70.5%)		
N1, N2	27(33.3%)	33(25.6%)		
**M stage**			0.427	0.632
M0	78(96.3%)	123(95.3%)		
M1	0(0%)	1(0.8%)		
**Surgical margin**			0.434	0.613
R0	67(82.7%)	100(77.5%)		
R1, R2	13(16.0%)	26(20.2%)		

Statistical analyses were performed using Pearson's χ2 test. *P < 0.05.

**Table 2:** Univariate and multivariate analyses of prognostic factors in CCA patients.

Variable	Univariate analysis				Multivariate analysis		
	HR	95% CI	P value		HR	95% CI	P value
Sex	0.704	0.507-0.978	**0.037**		0.699	0.474-1.031	0.071
Age	1.288	0.931-1.782	0.127				
Tumor size (cm)	1.505	1.025-2.208	**0.037**		1.517	1.017-2.263	**0.041**
Differentiation	1.590	1.157-2.185	**0.004**		1.221	0.841-1.775	0.294
Tumor Location	0.812	0.593-1.112	0.194				
Perineural invasion	1.127	0.810-1.568	0.477				
R0 resection	1.556	1.062-2.280	**0.023**		1.328	0.852-2.070	0.211
T stage	1.184	0.854-1.641	0.311				
N stage	1.929	1.392-2.674	**<0.001**		2.143	1.474-3.115	**<0.001**
M stage	1.699	0.027-12.197	0.598				
USP21 Expression	1.612	1.162-2.235	**0.004**		1.833	1.237-2.717	**0.003**

^*^P < 0.05

## Abbreviations

ACSS2: Acetyl-CoA synthetase 2
ALDOC: Aldolase c
CCA: Cholangiocarcinoma
CCK-8: Cell counting Kit-8
Co-IP: Co-immunoprecipitation
DFS: Disease-free survival
DUBs: Deubiquitinating enzymes
EDU: 5-ethynyl-2'-deoxyuridine
ENO1: Alpha-Enolase 1
ENO2: Enolase 2
ENO3: Enolase 3
FOXD1: Forkhead box D1
GEM: Gemcitabine
GEO: The Gene Expression Omnibus
HIF1A: Hypoxia-inducible factor -1a
HSP90: Heat-shock protein 90
IF: Immunofluorescence
IHC: Immunohistochemistry
IP/MS: Immunoprecipitation coupled mass spectrometry
KEGG: Kyoto Encyclopedia of Genes and Genomes
OS: Overall survival
TCGA: The Cancer Genome Atlas
Ub: Ubiquitin
USPs: Ubiquitin-specific proteases
USP21: Human ubiquitin-specific protease 21
