# Supplementary material for: USP21 deubiquitinates and stabilizes HSP90 and ENO1 to promote aerobic glycolysis and proliferation in cholangiocarcinoma
Source: Int J Biol Sci. 2024 Feb 4;20(4):1492–508. doi: 10.7150/ijbs.90774 (PMC10878141; doi:10.7150/ijbs.90774)
Supplement: Supplementary file 1 — Supplementary figures and tables. [file ijbsv20p1492s1.pdf]

USP21 deubiquitinates and stabilizes HSP90 and ENO1 to promote aerobic glycolysis and proliferation in cholangiocarcinoma.

Xiao Xu [1#], Yananlan Chen[1#], Shenye Shao[1#], Jifei Wang[1#], Jijun Shan[1], Yuming Wang[1], Yirui Wang[1], Jiang Chang[1], Tao Zhou[1], Ruixiang Chen[1], Shuochen Liu[1], Chang Li[1], Changxian Li[1,2*], Xiangcheng Li[1,2,3*]

[1] Hepatobiliary Center, The First Affiliated Hospital of Nanjing Medical University, Nanjing, China.

[2] Key Laboratory for Liver Transplantation, Chinese Academy of Medical Sciences, NHC Key Laboratory of Living Donor Liver Transplantation (Nanjing Medical University), Nanjing, Jiangsu Province, China.

[3] The Affiliated Wuxi People's Hospital of Nanjing Medical University, Wuxi People's Hospital, Wuxi Medical Center, Nanjing Medical University

***Correspondence:**
*Xiangcheng Li, M.D., Ph.D., Hepatobiliary Surgery Hepatobiliary Center, The First Affiliated Hospital of Nanjing Medical University, 300 Guangzhou Road, Nanjing, Jiangsu Province, China

Tel (Fax): 86-25-6830-3226
drxcli@njmu.edu.cn

*Changxian Li, M.D., Ph.D., Hepatobiliary Surgery Hepatobiliary Center, The First Affiliated Hospital of Nanjing Medical University, 300 Guangzhou Road, Nanjing, Jiangsu Province, China

Tel (Fax): 86-25-6830-3225
drlicx@njmu.edu.cn

# These authors contributed equally to this work.

Supplementary Figures:

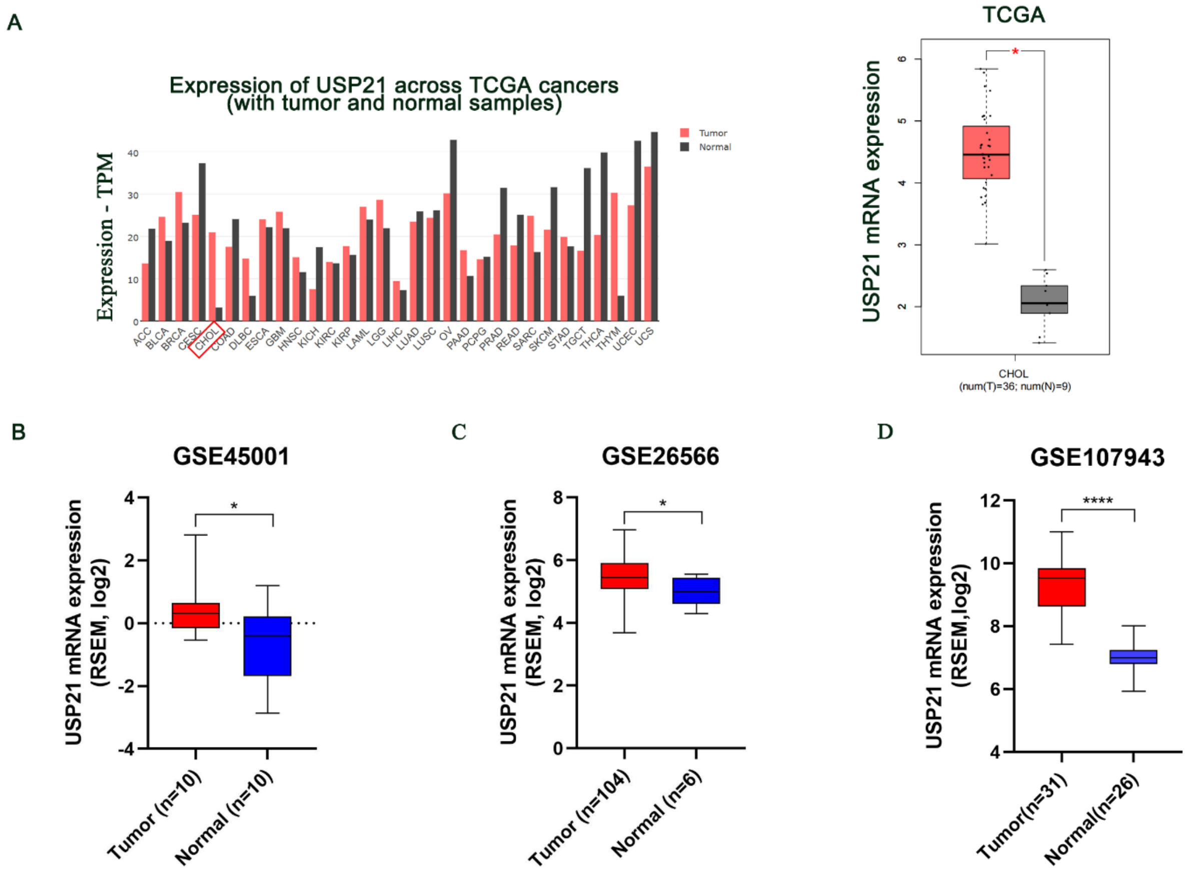

Supplementary Figure 1 USP21 was up-regulated in CCA tissues. (A) Analysis of USP21 mRNA expression levels in TCGA database. (B-C) Analysis of USP21 mRNA expression levels in the GSE45001 and GSE26566 databases. (D) Analysis of USP21 mRNA expression levels in the GSE107943 database. **P* < 0.05, ** *P* < 0.01, *** *P* < 0.001.

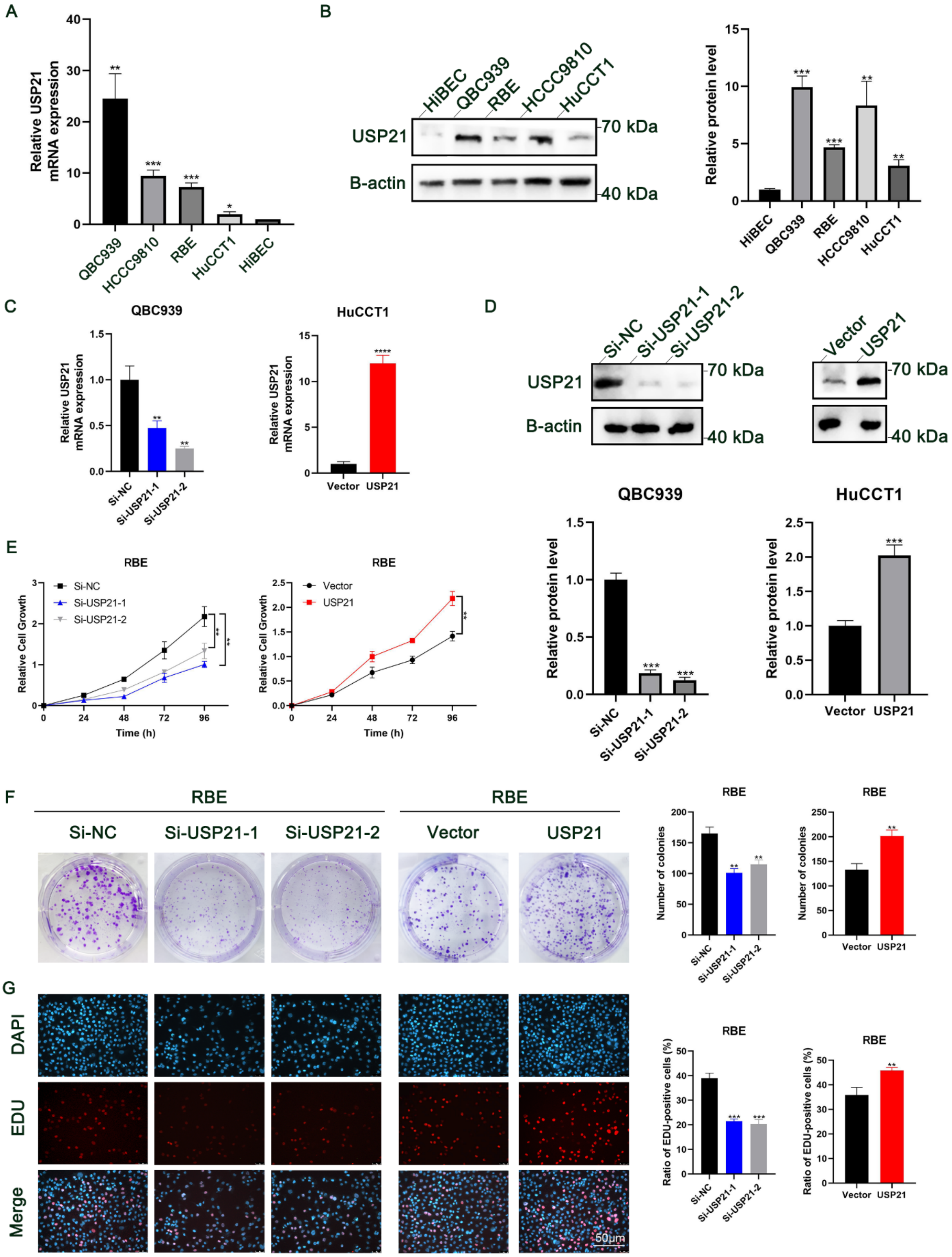

Supplementary Figure 2 USP21 transfection efficiency was verified in CCA cells. (A-B) The expression levels of USP21 mRNA and protein in CCA cell lines and normal bile duct epithelial cell lines were detected by RT-qPCR and western blotting. (C-D) The transfection efficiency was

confirmed using RT-qPCR and western blotting(C-D). CCK8 assays (E), plate clone formation assays (F), and EDU staining assays (G) indicated that USP21 promoted the proliferation of CCA cells. *$P < 0.05$, ** $P < 0.01$, *** $P < 0.001$.

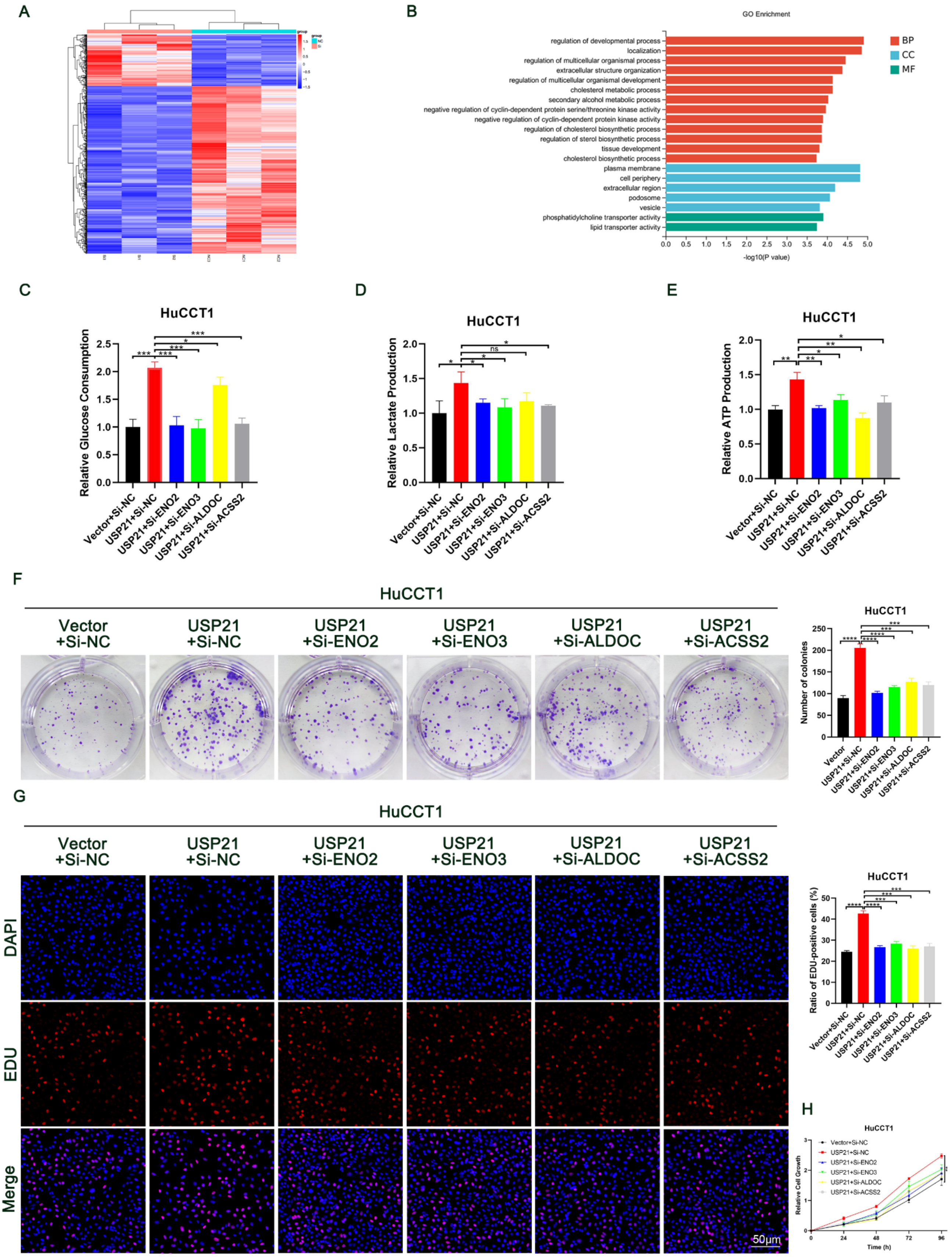
A
B
GO Enrichment
regulation of developmental process
localization
regulation of multicellular organismal process
extracellular structure organization
regulation of multicellular organismal development
cholesterol metabolic process
secondary alcohol metabolic process
negative regulation of cyclin-dependent protein serine/threonine kinase activity
negative regulation of cyclin-dependent protein kinase activity
regulation of cholesterol biosynthetic process
regulation of sterol biosynthetic process
tissue development
cholesterol biosynthetic process
plasma membrane
cell periphery
extracellular region
podosome
vesicle
phosphatidylcholine transporter activity
lipid transporter activity
-log10(P value)
BP
CC
MF
C
HuCCT1
Relative Glucose Consumption
D
HuCCT1
Relative Lactate Production
E
HuCCT1
Relative ATP Production
Vector+Si-NC
USP21+Si-NC
USP21+Si-ENO2
USP21+Si-ENO3
USP21+Si-ALDOC
USP21+Si-ACSS2
F
HuCCT1
Vector +Si-NC
USP21 +Si-NC
USP21 +Si-ENO2
USP21 +Si-ENO3
USP21 +Si-ALDOC
USP21 +Si-ACSS2
Number of colonies
G
HuCCT1
DAPI
EDU
Merge
50μm
Ratio of EDU-positive cells (%)
H
HuCCT1
Relative Cell Growth
Time (h)

Supplementary Figure 3 USP21 promotes CCA proliferation by enhancing aerobic glycolysis. (A) Heatmap of differentially expressed genes, which transfected in NC sequence compared with Si-USP21 sequence. (B) GO analysis of downregulated genes in USP21-KD QBC939 cells. ENO2, ENO3, ALDOC, and ACSS2 knockdown reversed USP21-mediated increases in glucose consumption (C), lactate production (D), and cellular ATP levels (E) in HuCCT1 cells. (F) Colony formation assays, (G) Edu staining assays, and (H) CCK8 assays were performed to determine the effects of glycolytic genes on USP21-mediated cell proliferation. *$P < 0.05$, ** $P < 0.01$, *** $P < 0.001$.

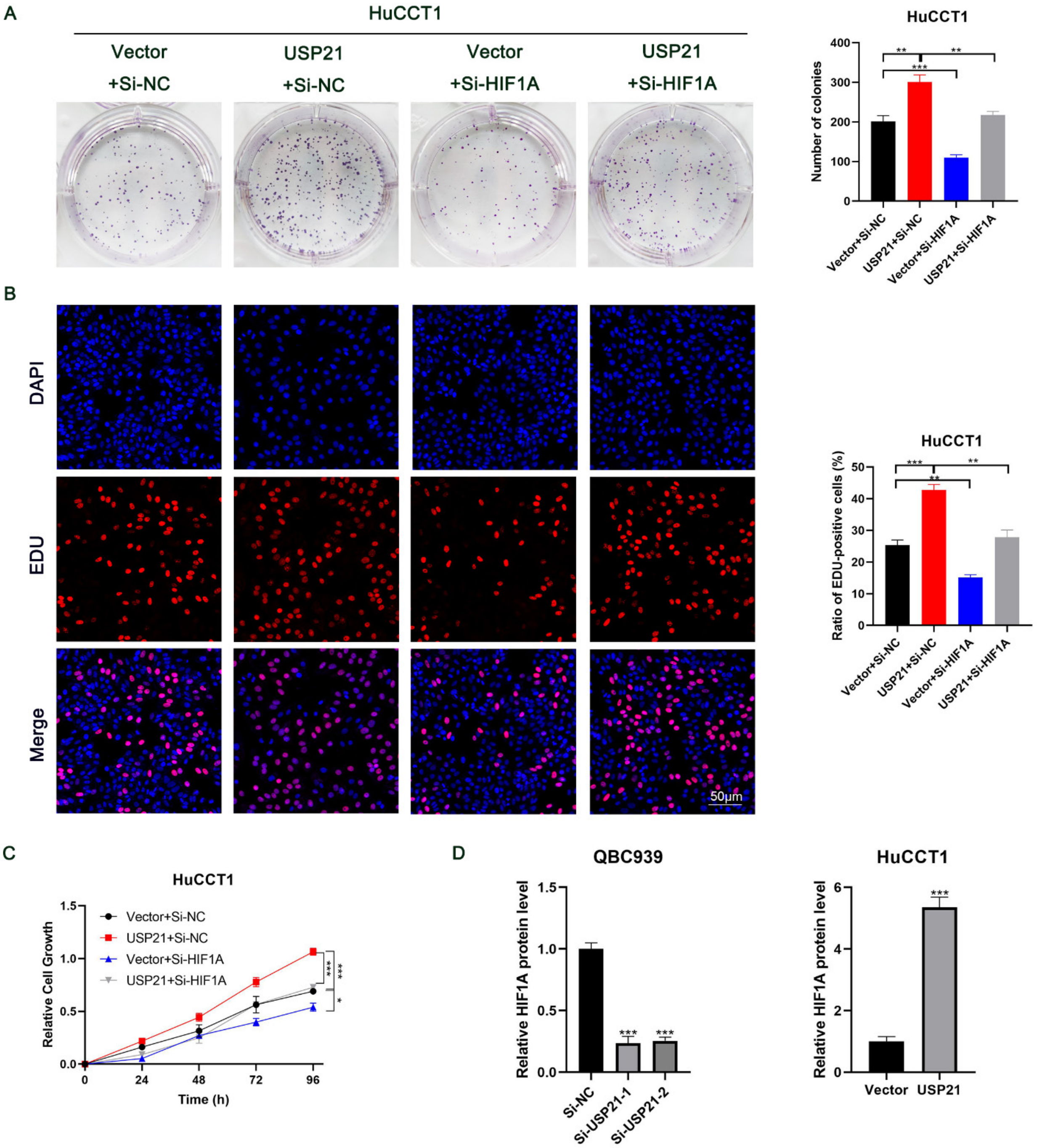

Supplementary Figure 4 USP21 promotes aerobic glycolysis and tumor growth in a HIF1A-dependent manner. Functional assays containing Plate clone formation assays (A), Edu staining assays (B), and CCK8 assays (C) were performed to detect the rescued effect of HIF1A on USP21. (D) Grayscale statistical charts for western blots in Figure 3J. *$P < 0.05$, ** $P < 0.01$, *** $P < 0.001$.

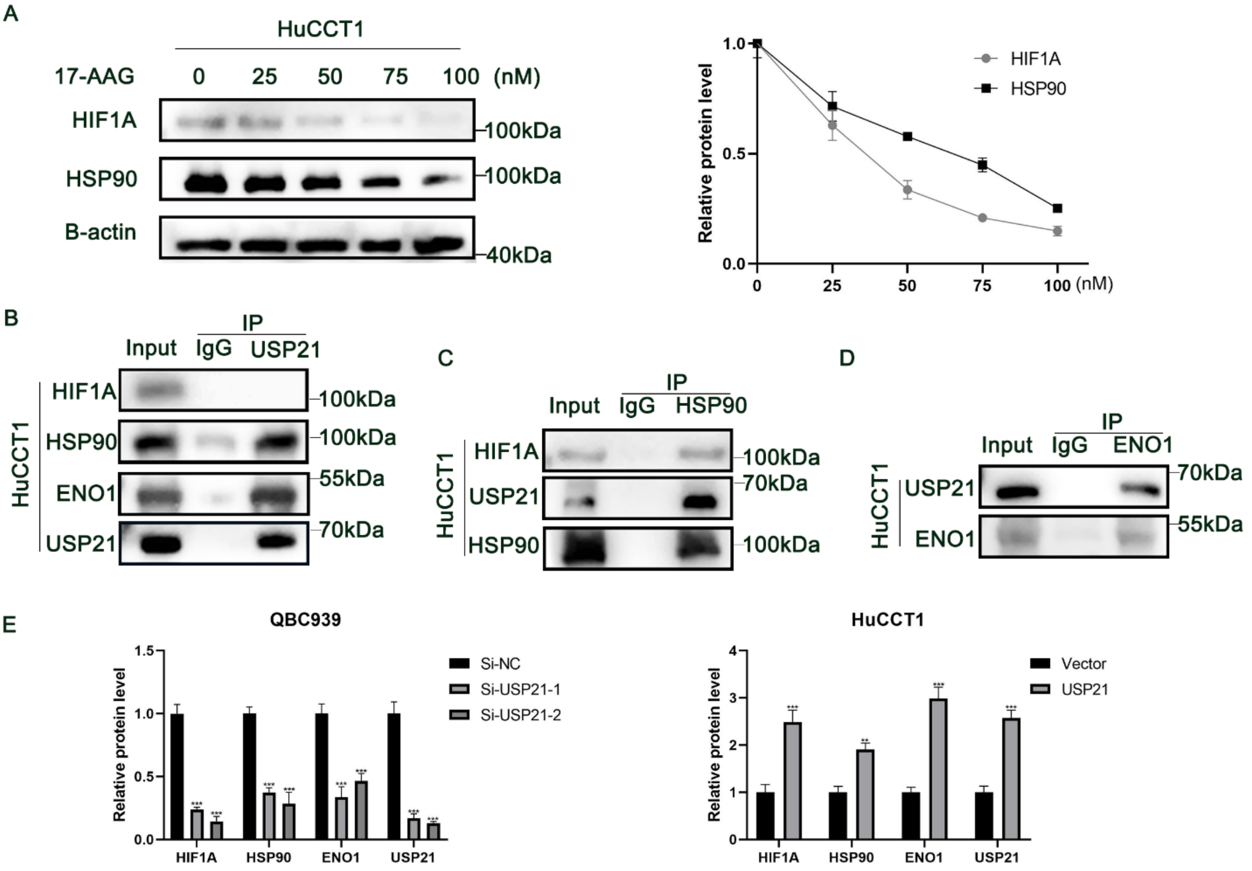

Supplementary Figure 5 USP21 interacted with HSP90 and ENO1. (A) HIF1A expression levels were detected in HuCCT1 cells cultured in different concentrations of 17-AAG for 24 h by Western blotting. (B) Co-IP was performed to confirm the binding of HSP90 and ENO1 to USP21 in HuCCT1 cells. (C-D) Immunoprecipitation assays were performed in HuCCT1 cells to examine the endogenic interaction among HIF1A, HSP90, ENO1, and USP21. (E) Grayscale statistical charts for western blots in Figure 4I. *$P < 0.05$, ** $P < 0.01$, *** $P < 0.001$.

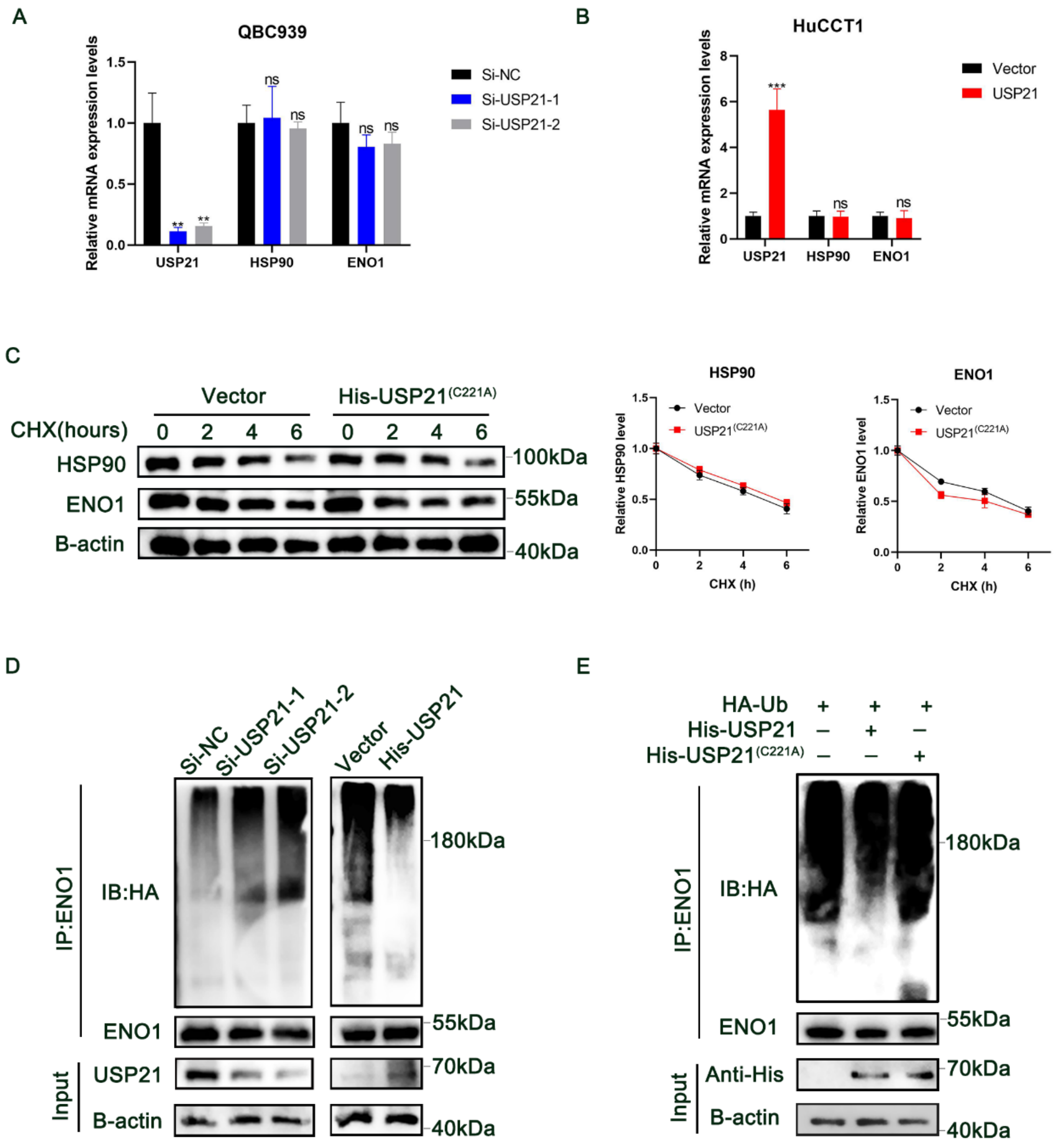

Supplementary Figure 6 USP21 inhibited ENO1 ubiquitination and degradation. (A-B) The relative mRNA expression levels of HSP90 and ENO1 were examined by RT-qPCR. (C) HSP90 and ENO1 protein levels in CCA cells transfected with USP21 (C221A) were measured by western blotting in the absence and presence of cycloheximide (CHX, 10 μg/mL) for a specified time. (D) Lysates from CCA cells treated with MG132 before collecting were subjected to immunoprecipitation and detected with the indicated antibodies. (E) The lysates of HEK 293T cells transfected with HA-Ub as well as His-labeled USP21 (WT) or His-labeled USP21 (C221A), were immunoprecipitated and subjected to anti-HA and anti-ENO1 immunoblotting.

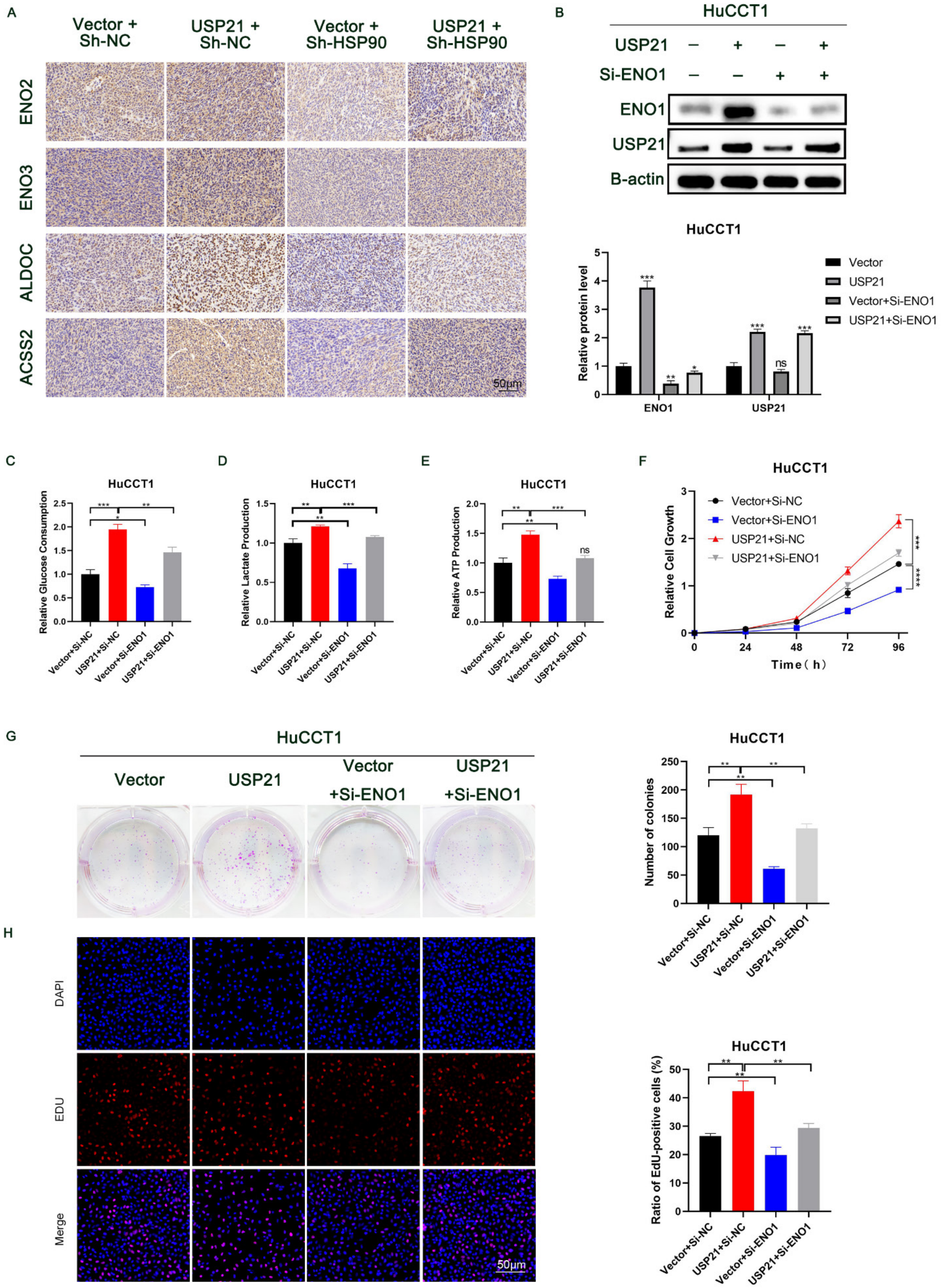

Supplementary Figure 7 USP21 promoted aerobic glycolysis and tumor growth by increasing

ENO1 levels. (A) The expression levels of ENO2, ENO3, ALDOC, and ACSS2 in nude mice-derived xenograft tumors were determined by IHC. (B) USP21 overexpression HuCCT1 cells were treated with Si-ENO1, and the expression levels of USP21 and ENO1 were verified by western blotting. ENO1 knockdown reversed USP21-mediated increases in glucose consumption (C), lactate production (D), and cellular ATP levels (E) in HuCCT1 cells. Functional assays containing CCK8 assays (F), Plate clone formation assays (G), and Edu staining assays (H) were performed to detect the rescued effect of ENO1 on USP21. *$P < 0.05$, ** $P < 0.01$, *** $P < 0.001$.

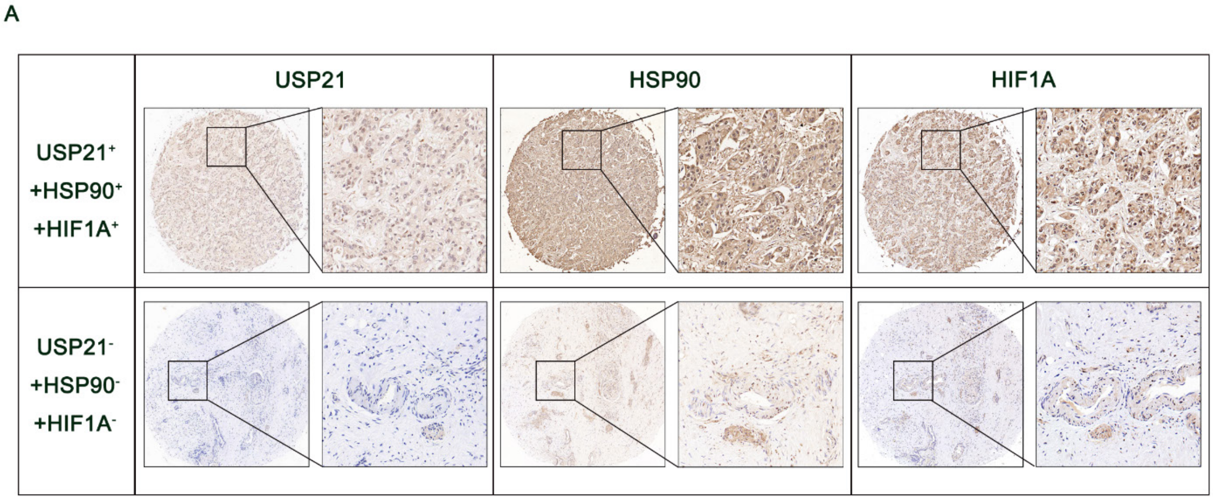

Supplementary Figure 8 High HSP90, HIF1A, and USP21 expression were associated with poor prognosis. (A) Representative images of high/low expression of USP21, HSP90, and HIF1A in tumors.

Supplementary materials and methods:

Cell culture

Human CCA cell lines RBE, HCCC9810, QBC939, HuCCT1, and human bile duct epithelial cell HiBEC were purchased from the Chinese National Human Genome Center (Shanghai, China) and cultured in Dulbecco's modified Eagle's medium (Gibco, USA) supplemented with 10% fetal bovine serum (FBS) (Gibco, Grand Island, NY, USA) and 1% antibiotics (penicillin/ streptomycin; Gibco). 293T cells were purchased from ATCC and cultured in DMEM with the same condition as CCA cell lines. All cells were maintained at 37°C in a humidified atmosphere of 5% CO2.

Animal Experiment

Six-week-old male BALB/c nude mice (GemPharmatech Co., Ltd., China) were housed under standard laboratory conditions. They were provided with adequate food and water, ambient relative humidity of 50–60%, controlled temperature of 22–26 °C, and a light/dark cycle of 12 h. QBC939 and HuCCT1 cells (5×10^6 cells/100 μL) were injected into the flanks of BALB/c nude mice to perform the subcutaneous tumorigenesis assay. All mice were examined weekly for tumor growth and health status. Tumors were harvested after 4 weeks and their volumes were calculated according to the following formula: volume = $width^2 \times length/2$. For gemcitabine efficacy analysis, 5×10^6 cells mentioned above were inoculated subcutaneously in mice (n=5 in each group). After 10 days of inoculation, the mice were intraperitoneally injected with PBS or gemcitabine (20 mg/kg) five times at 4-day intervals. Tumors were harvested 4 weeks after inoculation and then fixed in 4% formaldehyde.

Colony formation assay

CCA cells were seeded in a 6-well plate (500 cells/well) and cultured in 4mL of the medium at 37°C for 2 weeks. The proliferating colonies were then fixed with 4% paraformaldehyde (Beyotime, China) and stained with crystal violet (Beyotime, China). Finally, the visible colonies were photographed and counted.

Cell counting Kit-8 (CCK-8) assay

CCK8 assay kit (Dojindo, Japan) was used to assess cell proliferation. Cells were seeded in 96-well plates at 1×103 cells/well. On each day of the subsequent 5 days, 100μ medium containing 10μ CCK8 reagent was added into each well. The absorbance at 450nm was measured with a microplate reader.

EDU assay

EDU cell proliferation kit (Beyotime, China) was used for EDU assay to assess cell proliferation. Cells were planted in a 24-well plate and cultured for 24h, followed by 2h incubation with 10μM EDU medium. The cells were fixed in 4% paraformaldehyde for 15min and permeabilized with 0.3% Triton for 15 min. Sequentially the cells were stained with Alexa Fluor 555 azide for 30 min and DAPI Staining Solution (Beyotime, China) for 30 min in the dark. The cells were photographed under a fluorescence microscope (Olympus, Japan).

Quantitative PCR analysis (qRT-PCR)

Total RNA from CCA cells was extracted using a total RNA isolation kit (Vazyme, Nanjing, China) and cDNA was synthesized using PrimeScript RT reagents (Vazyme). RT-qPCR was performed with AceQ qPCR SYBR Green Master Mix (Vazyme, China). RNA relative expression was calculated using the 2-△△CT method with β-actin as an endogenous control. Primers used in this study are listed below.

Western blotting

Total protein was extracted from cell lines with NP-40 Lysis Buffer (Beyotime, China) supplemented with 1mM PMSF and 1mM Phosphatase inhibitor. The protein samples were transferred to

polyvinylidene difluoride (PVDF) membranes (Roche, Shanghai, China) using 10% SDS-polyacrylamide gels (Beyotime, Shanghai, China). The PVDF membranes were blocked in blocking buffer (Beyotime) for 20 min and overnight incubated with the primary antibody in dilution buffer at 4 °C. Subsequently, the PVDF membrane was cultured with the corresponding secondary antibody for 2 h. Finally, the protein bands were visualized using an ECL kit (YEASEN, Shanghai, China) and a chemiluminescent gel imaging system (Vilber, Paris, France). The related antibodies were listed at the end of this file.

Knockdown of Target Genes

Small interfering RNA (siRNA) targeting USP21, ENO1, and HIF1A were ordered from GeneChem (Shanghai, China). The cells were transfected with siRNA using lipofectamine 3000 (Invitrogen, USA) according to the manufacturer's instructions. The oligonucleotide sequences were listed below.

Plasmids and adenoviral infection

Cells were infected with adenovirus shRNA-control (sh-NC), adenovirus shRNA- USP21, or shRNA-HSP90 (sh-USP21, or sh-HSP9), adenovirus vector (Vector), and adenovirus USP21, or USP21$^{(C221A)}$ (USP21, or USP21$^{(C221A)}$). Full-length sequences for human USP21 and ubiquitin were subcloned into the EcoRI and NotI sites of His-, and HA-tagged pcDNA3.1 vectors (Thermo Fisher Scientific).

Measurements of glucose consumption and lactate production.

Briefly, CCA cells were seeded in 6-well plates (2×10^5 cells/well), replaced with 2 ml serum-free DMEM, and the medium was collected 24h later to determine lactate production and glucose consumption respectively. Lactate and glucose levels in the culture medium were determined using kits obtained from Beyotime (S0201S) and Elabscience (E-BC-K044-S), according to the manufacturer's instructions. Glucose consumption was calculated by the difference in glucose concentration in the medium before and after cell incubation.

Determination of ATP content

CCA cell ATP content was determined by an Enhanced ATP Assay Kit (Beyotime, China) according to the manufacturer's instructions, and the results are shown in arbitrary units.

Reagents

**Name**	**Cat no.**	**Supplier**
Cycloheximide (CHX)	HY-12320	MCE
MG132	HY-13259	MCE
17-AAG	HY-10211	MCE
Gemcitabine	HY-17026	MCE

Sequences of primers and siRNAs

1. Primers used in RT-qPCR analysis

**Gene**	**Forward Primer**	**Reverse Primer**
USP21	CAGGTCTGCCTGATGAACGG	GCTAAGTTGGTCCGAGATGGG
ENO2	AGCCTCTACGGGCATCTATGA	TTCTCAGTCCCATCCAACTCC
ENO3	GGCTGGTTACCCAGACAAGG	TCGTACTTCCCATTGCGATAGAA
ALDOC	ATGCCTCACTCGTACCCAG	TTTCCACCCCAATTTGGCTCA
ACSS2	AAAGGAGCAACTACCAACATCTG	GCTGAACTGACACACTTGGAC
HIF1A	GAACGTCGAAAAGAAAAGTCTCG	CCTTATCAAGATGCGAACTCACA
B-actin	CATGTACGTTGCTATCCAGGC	CTCCTTAATGTCACGCACGAT

2. siRNAs

**Gene**	**Sense (5'-3')**
USP21#1	TCACTAAGGAAGAAGAGCT
USP21#2	AACCTAATGTGGAAACGTT
HIF1A	CAUGAAAGCACAGAUGAAUTT
ENO1	CGAGAUGGAUGGAACAGAA
ENO2	CGUUCUGAACGUCUGGCUAAATT
ENO3	CCAACAUCCUGGAGAACAATT
ALDOC	GCAGCACAGTCACTCTACATT

Primary antibodies used in this study.

Antibody	Company (Cat. No.)
USP21	Santa Cruz Biotechnology (sc-515911)
ENO1	Proteintech (11204-1-AP)
ENO2	Proteintech (66150-1-Ig)
ENO3	Proteintech (55234-1-AP)
ALDOC	Abcam (ab302952)
ACSS2	Abcam (ab133664)
HSP90	Proteintech (13171-1-AP)
HIF1A	Abcam (ab51608)
Anti-His	Proteintech (66005-1-Ig)
Anti-HA	Abcam (ab236632)
PCNA	Proteintech (10205-2-AP)
Ki-67	Proteintech (27309-1-AP)
Gamma H2A.X	Abcam (ab81299)
B-actin	Proteintech (20536-1-AP)

Supplementary Tables:

Supplemental Table. 1 Association of HSP90 expression with clinicopathological features of CCA.

Clinicopathological features	HSP90 expression		P value	χ2
	downregulated	upregulated		
**All cases**	91(43.3%)	119(56.7%)		
**Gender**			0.547	0.363
Male	61(67.0%)	75(63.0%)		
Female	30(33.0%)	44(37.0%)		
**Age**			0.622	0.242
≤60	49(53.8%)	60(50.4%)		
＞60	42(46.2%)	59(49.6%)		
**Diameter(cm)**			0.738	0.111
≤2.5	23(25.2%)	25(21.0%)		
＞2.5	55(60.4%)	67(56.3%)		
**Location**			0.853	0.034
Intrahepatic	46(50.5%)	60(50.4%)		
Perihilar	40(44.0%)	55(46.2%)		
**Histological grade**			0.208	1.586
I/I-II/II	46(50.5%)	50(42.0%)		
II-III/III	39(42.9%)	61(51.3%)		
**Perineural invasion**			0.993	0.001
Absent	37(40.7%)	51(42.9%)		
Present	40(44.0%)	55(46.2%)		
**Tumor thrombus**			0.779	0.079
Absent	74(81.3%)	95(79.8%)		
Present	14(15.4%)	20(16.8%)		
**T stage**			0.993	0.001
Tis-T1	32(35.2%)	42(35.3%)		
T2-T4	55(60.4%)	72(60.5%)		
**N stage**			0.399	0.712
N0	64(70.3%)	79(66.4%)		
N1, N2	23(25.3%)	37(31.1%)		
**M stage**			0.383	0.760
M0	87(95.6%)	114(95.8%)		
M1	0(0%)	1(0.01%)		
**Surgical margin**			**0.014**	**6.047**
R0	79(86.8%)	88(73.9%)		
R1，R2	10(11.0%)	29(24.4%)		

Statistical analyses were performed using Pearson's χ2 test. *$P < 0.05$.

Supplemental Table. 2 Univariate and multivariate analyses of prognostic factors in CCA patients.

Variable	Univariate analysis			Multivariate analysis		
	HR	95% CI	P value	HR	95% CI	P value
Sex	0.704	0.507-0.978	**0.037**	0.713	0.482-1.054	0.089
Age	1.288	0.931-1.782	0.127			
Tumor size (cm)	1.505	1.025-2.208	**0.037**	1.440	0.965-2.149	0.074
Differentiation	1.590	1.157-2.185	**0.004**	1.242	0.858-1.802	0.255
Tumor Location	0.812	0.593-1.112	0.194			
Perineural invasion	1.127	0.810-1.568	0.477			
R0 resection	1.556	1.062-2.280	**0.023**	1.148	0.733-1.798	0.546
T stage	1.184	0.854-1.641	0.311			
N stage	1.929	1.392-2.674	**<0.001**	1.940	1.341-2.805	**<0.001**
M stage	1.699	0.027-12.197	0.598			
HSP90 Expression	1.768	1.289-2.423	**<0.001**	1.487	1.025-2.158	**0.037**

*$P < 0.05$

Supplemental Table. 3 Association of HIF1A expression with clinicopathological features of CCA.

Clinicopathological features	HIF1A expression		P value	χ2
	downregulated	upregulated		
**All cases**	60(28.6%)	150(71.4%)		
**Gender**			0.493	0.469
Male	41(68.3%)	95(63.3%)		
Female	19(31.7%)	55(36.7%)		
**Age**			0.238	1.391
≤60	35(58.3%)	74(49.3%)		
＞60	25(41.7%)	76(50.7%)		
**Diameter(cm)**			0.068	3.333
≤2.5	19(31.7%)	29(19.3%)		
＞2.5	31(51.7%)	91(60.7%)		
**Location**			0.154	2.037
Intrahepatic	24(40.0%)	82(54.7%)		
Perihilar	30(50.0%)	65(43.4%)		
**Histological grade**			**0.017**	**5.735**
I/I-II/II	35(58.3%)	61(40.7%)		
II-III/III	21(35.0%)	79(52.7%)		
**Perineural invasion**			0.602	0.273
Absent	22(36.7%)	66(44.0%)		
Present	27(45.0%)	68(45.3%)		
**Tumor thrombus**			0.138	2.201
Absent	51(85.0%)	118(78.7%)		
Present	6(10.0%)	28(18.7%)		
**T stage**			0.535	0.385
Tis-T1	18(30.0%)	56(37.3%)		
T2-T4	36(60.0%)	91(60.7%)		
**N stage**			0.664	0.189
N0	40(66.7%)	103(68.7%)		
N1, N2	15(25.0%)	45(30.0%)		
**M stage**			0.545	0.367
M0	54(90.0%)	147(98.0%)		
M1	0(0%)	1(0.7%)		
**Surgical margin**			0.810	0.058
R0	46(76.7%)	121(80.7%)		
R1，R2	10(16.7%)	29(19.3%)		

Statistical analyses were performed using Pearson's χ2 test. *$P < 0.05$.

Supplemental Table. 4 Univariate and multivariate analyses of prognostic factors in CCA patients.

Variable	Univariate analysis			Multivariate analysis		
	HR	95% CI	P value	HR	95% CI	P value
Sex	0.704	0.507-0.978	**0.037**	0.689	0.466-1.020	0.063
Age	1.288	0.931-1.782	0.127			
Tumor size (cm)	1.505	1.025-2.208	**0.037**	1.352	0.904-2.022	0.142
Differentiation	1.590	1.157-2.185	**0.004**	1.242	0.858-1.797	0.252
Tumor Location	0.812	0.593-1.112	0.194			
Perineural invasion	1.127	0.810-1.568	0.477			
R0 resection	1.556	1.062-2.280	**0.023**	1.218	0.783-1.218	0.382
T stage	1.184	0.854-1.641	0.311			
N stage	1.929	1.392-2.674	**<0.001**	1.953	1.351-2.823	**<0.001**
M stage	1.699	0.027-12.197	0.598			
HIF1A Expression	1.696	1.186-2.245	**0.004**	1.638	1.080-2.483	**0.020**

$^{*}P < 0.05$

Supplemental Table. 5 Correlation between USP21 and HSP90 and HIF1A protein expression.

	USP21 expression			
	High ($USP21^{+}$)	Low ($USP21^{-}$)	P value	$\chi2$
**Total**	129	81		
**HSP90**			**0.048**	**3.897**
High ($HSP90^{+}$)	80	39		
Low ($HSP90^{-}$)	49	42		
**HIF1A**			**0.031**	**4.630**
High ($HIF1A^{+}$)	99	51		
Low ($HIF1A^{-}$)	30	30		

$^{*}P < 0.05$
